# Cross‐Scale Decoupling Kinetic Processes in Lithium‐Ion Batteries Using the Multi‐Dimensional Distribution of Relaxation Time

**DOI:** 10.1002/advs.202406934

**Published:** 2024-10-08

**Authors:** Xue Cai, Caiping Zhang, Haijun Ruan, Zeping Chen, Linjing Zhang, Dirk Uwe Sauer, Weihan Li

**Affiliations:** ^1^ National Active Distribution Network Technology Research Center Beijing Jiaotong University Beijing 100044 China; ^2^ Center for Ageing, Reliability and Lifetime Prediction of Electrochemical and Power Electronic Systems (CARL) Campus‐Boulevard 89 52074 Aachen Germany; ^3^ Centre for E‐Mobility and Clean Growth Research Coventry University Coventry CV1 5FB UK; ^4^ Dyson School of Design Engineering Imperial College London London SW7 2AZ UK; ^5^ Helmholtz Institute Münster (HI MS) IEK‐12 Forschungszentrum Jülich Germany

**Keywords:** cross‐scale identification, distribution of relaxation time, kinetic processes decoupling, lithium‐ion batteries, reconstructed interfacial impedance

## Abstract

To non‐destructively resolve and diagnose the degradation mechanisms of lithium‐ion batteries (LIBs), it is necessary to cross‐scale decouple complex kinetic processes through the distribution of relaxation times (DRT). However, LIBs with low interfacial impedance render DRT unreliable without data processing and closed‐loop validation. This study proposes a hierarchical analytical framework to enhance timescale resolution and reduce uncertainty, including interfacial impedance reconstruction and multi‐dimensional DRT analysis. Interfacial impedance is reconstructed by eliminating simulated inductive and diffusive impedance based on a high‐fidelity frequency‐domain model. Multi‐dimensional DRT decouples solid electrolyte interphase (SEI) and charge transfer (CT) processes by the reversibility of electrochemical reactions with state of charge (SOC) to characterize electrode kinetic evolution driven by SOC and temperature through timescales and peak area. The findings reveal that reconstructed impedance improves the accuracy of identified time constants by ≈20%. Cross‐scale DRT results reveal that SOCs below 10% at 25 °C effectively distinguish electrode kinetics due to the high correlation between cathodic CT and SOC. Kinetic metrics characterize that anodic SEI or CT are different control steps limiting the low‐temperature performance of different cells. This work underscores the potential of the proposed framework for non‐destructive diagnostics of kinetic evolution.

## Graphical abstract

Xue et al. propose a hierarchical framework for cross‐scale decoupling of complex kinetic processes with inductance and diffusion processes rooted in milliohm‐level interfacial impedance. This approach encompasses data pre‐processing, interfacial impedance reconstruction, and multi‐dimensional distribution of relaxation time (DRT) analysis, improving DRT accuracy by 20% and further illuminating the potential of DRT in cross‐scale nondestructive diagnosis.

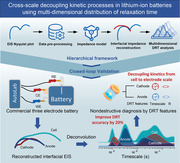

## 1 Introduction

Recent advancements in electric vehicles and renewable energy are crucial for achieving carbon peaking and neutrality goals.^[^ ^1^, ^2^ ^]^ Central to these advancements is the development of highly integrated and reliable energy storage systems. Lithium‐ion batteries (LIBs), known for their high energy/power density and cost‐effectiveness,^[^ ^3^, ^4^ ^]^ have been the predominant technology for such systems. Ensuring the operational reliability and safety of LIBs under varying conditions, such as temperature,^[^ ^5^, ^6^ ^]^ pressure,^[^ ^7^, ^8^, ^9^ ^]^ and aging,^[^ ^10^, ^11^, ^12^ ^]^ necessitates a deep understanding of the complex electrochemical processes occurring within LIBs.

Non‐destructive diagnostic technologies, such as electrochemical impedance spectroscopy (EIS)^[^ ^5^, ^13^, ^14^ ^]^ and distribution of relaxation time (DRT),^[^ ^15^, ^16^, ^17^, ^18^, ^19^ ^]^ provide profound insight into the kinetic evolution of electrochemical processes under specific stress. These tools provide valuable physical information essential for optimizing the performance and safety monitoring of LIBs. However, while model‐based EIS relies on researchers' knowledge of parameter boundaries to avoid uncertain diagnostics, particularly in unexplored electrochemical systems,^[^ ^20^ ^]^ timescale‐based DRT can identify characteristic times of electrochemical processes from multi‐peak plots without prior knowledge.^[^ ^21^ ^]^ This capability aids in revealing internal electrochemical mechanisms through both direct feature extraction (e.g., height, location, and area)^[^ ^22^, ^23^, ^24^, ^25^, ^26^ ^]^ and indirect fitted parameters (e.g., equivalent and electrochemical impedance models).^[^ ^27^, ^28^, ^29^ ^]^

While the DRT technique offers valuable insights into the timescale identification of electrochemical reactions, achieving high precision in DRT remains a challenge, particularly in data processing and timescale validation. Often overlooked due to limited understanding of coupled electrochemical reactions, data processing is critical for LIBs with low interfacial EIS, known as LI‐LIBs. Our previous study^[^ ^30^ ^]^ revealed LI‐LIBs minimal semi‐circular arcs in Nyquist plots, indicating a negligible visible interfacial impedance in ≈0.5 mΩ ohmic resistance. With the market emphasizing high energy and power density, LI‐LIBs are increasingly prevalent. However, traditional DRT analysis, directly extracting part frequency (EPF) directly,^[^ ^31^, ^32^ ^]^ hasn't been successful in LI‐LIBs. Theoretically, resistor and constant phase elements (R/Q‐like elements), combined with radial basis functions,^[^ ^32^, ^33^ ^]^ could better represent interfacial processes than inductive and diffusive ones. The accuracy of DRT in LI‐LIBs is notably affected by process interactions, such as the coupling of interface reaction with high‐frequency (HF) cable inductance and low‐frequency (LF) diffusion kinetics. The former coupling depends on their relative magnitudes. At the same time, the impact of diffusion is quantifiable by the distance from knee points to the real axis, and it is controlled by differential voltage (DV).^[^ ^34^ ^]^ Previous studies, like Zhu et al.^[^ ^32^ ^]^ and Sophia et al.,^[^ ^10^, ^11^ ^]^ demonstrate that DRT analysis, conducted on pseudo‐interfacial EIS before the knee point, allows partial diffusion to blend into interfacial kinetics, with inductance similarly influencing results. Nevertheless, comprehensive discussions on effective data processing for interfacial impedance and factors influencing DRT outcomes are scarce. Thus, a generalized DRT analytical framework is urgently needed to enhance the accuracy of interfacial DRT results.

Moreover, while timescale‐based DRT facilitates the decoupling of kinetic processes, validating these identified timescales from cell to electrode scales remains a formidable challenge. Gathering cross‐scale information necessitates either disassembling or modifying commercial batteries, employing both in situ^[^ ^35^, ^36^, ^37^ ^]^ and ex situ^[^ ^10^, ^11^, ^32^, ^38^ ^]^ experimental methods. Ex situ approaches, such as half and symmetric cells, often introduce additional interference or practical difficulties. For instance, Li‐symmetric cells can obscure the DRT of charge transfer processes in the working electrode due to the influence of the Li counter electrode, potentially leading to erroneous conclusions, as highlighted by Sabet et al.^[^ ^11^ ^]^ Furthermore, assembling symmetric cells across various states of charge (SOCs) is time‐consuming and labor‐intensive, complicating the correlation between timescale and SOC at different scales. To overcome these limitations, the three‐electrode battery, an in situ method,^[^ ^37^ ^]^ has been extensively used to isolate kinetic processes^[^ ^32^ ^]^ and evaluate the electrode's contribution to cell impedance.^[^ ^28^ ^]^ Despite these advancements, the three‐electrode battery still faces challenges associated with the influence of inductive and diffusive processes on interfacial DRT.

Dimensional extension in DRT, initially introduced to incorporate temperature conditions, significantly enhances the precision and fidelity of DRT analysis.^[^ ^19^ ^]^ Activation energy, acting as a distinct signature, further refines the characterization of interfacial reactions,^[^ ^32^ ^]^ diminishing uncertainty. This framework has expanded to encompass diverse experimental conditions, spanning alterations in gas, electrolyte volume, and aging conditions.^[^ ^21^ ^]^ While artificial intelligence has been employed to tackle the complexity of large‐scale experiments, its efficacy is hindered by a lack of comprehensive physical understanding. Consequently, multi‐dimensional DRT has emerged as a pivotal tool for monitoring battery states and identifying stress‐driven control steps, aiding in regulating new cell chemistry. Within the realm of multi‐dimensional DRT, the reversibility of processes serves as a key criterion for distinguishing different kinetics.^[^ ^21^ ^]^ For example, interfacial charge transfer (CT) is a dynamic process influenced by phase composition and ionic concentration,^[^ ^39^ ^]^ contrasting with the static nature of ionic conduction within the solid‐electrolyte interphase (SEI)^[^ ^21^ ^]^ and responses within solid‐state electrolytes.^[^ ^40^ ^]^ Leveraging comprehensive physical insights can refine timescale identification and polarization characterization, revealing key control steps like anodic SEI formation and CT at low temperatures. Nonetheless, a comprehensive discourse is yet to materialize on implementing kinetic processes decoupling and validation, guided by multi‐dimensional DRT and particularly tailored for LI‐LIBs. This gap underscores the imperative for a generalized, close‐loop analytical framework for LI‐LIBs, aimed at precise timescale identification and validation of decoupled kinetic processes.

**Figure 1:**
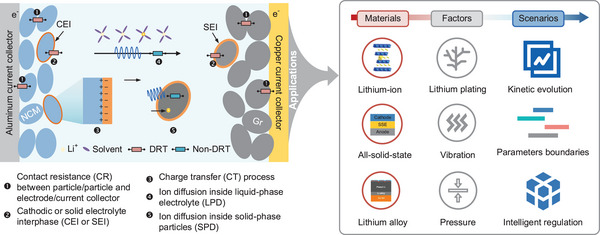
Mechanisms of timescale identification and their potential applications.

To address the abovementioned problems in decoupling complex kinetic processes by timescale identification and validation, we propose a hierarchical analytical framework using the DRT in retrofitted commercial batteries featuring a reference electrode, namely three‐electrode batteries. This framework incorporates reconstructed interfacial impedance and multi‐dimensional DRT analysis. We developed an integrated model combining the advantages of both thermodynamic and kinetic models to reconstruct open‐circuit voltage (OCV) and interfacial impedance. Correcting the impedance data based on reconstructed OCV, we accounted for OCV shifts akin to voltage amplitudes in the LF region. Employing a multi‐step and multi‐objective (MSMO) optimization approach, we identified impedance model parameters from modified data. By extending the inductive and diffusive model across frequencies, we reconstructed pure interfacial impedance from overall impedance, shedding light on how inductance and diffusion processes influence interfacial DRT through numerical simulation. Focusing on reconstructed interfacial impedance datasets under various conditions, we implemented multi‐dimensional DRT analysis to identify electrochemical timescales by assessing the reversibility of kinetics with SOC. This analysis establishes links between DRT features and kinetic evolution, unveiling effective conditions for distinguishing electrode CT timescales from cell‐level dynamics and identifying the key controlling steps restricting battery low temperature (LT) performance. Our work showcases the potential of the proposed framework to reduce uncertainties and enhance DRT resolution, thus enabling non‐destructive analysis of kinetics driven by various factors and expanding the functional application for new electrochemistry, as shown in **Figure** **1**.

## 2 Methodology

### 2.1 Mathematical Theory of DRT

Identifying electrochemical information about individual electrodes from cell‐level impedance data is quite challenging, primarily due to the concealment and overlap of key impedance features resulting from the similar timescales of electrochemical reactions observed in Nyquist plots. Nevertheless, the DRT technique, designed for unraveling EIS data, holds significant promise for discerning kinetics timescales. From equivalent circuit models (ECMs), a single DRT peak is conceptualized as an *R*/*Q* network, achieved through the aggregation and integration of multiple radial basis functions (RBFs) of Gaussian distribution.^[^ ^31^ ^]^ In contrast to an ideal R/C network, the *R*/*Q* network offers a more accurate portrayal of the genuine interfacial dynamics associated with double‐layer capacitance.

(1) $$Z_{RQ}\left(\omega \right)=\frac{R}{1+{\left(j\omega RC\right)}^{\alpha }}=\frac{R}{1+{\left(j\omega \tau_{c}\right)}^{\alpha }}$$

where *α* represents a constant ranging from 0 to 1, indicating the fractional double‐layer capacitance, *ω* denotes the angular frequency of the current. *Q* equals *C* ^α^, signifying a constant phase element. *τ*_c_ is the characteristic time constant. In DRT, each *R*/*Q* network can be represented as a series of RBFs featuring independent time constants^[^ ^33^ ^]^ ranging from *τ*_1_ to *τ*_N_.

(2) $$Z_{\mathrm{DRT}}\left(\omega \right)=\sum_{i=1}^{N}\frac{R_{i}}{1+{\left(j\omega \tau_{i}\right)}^{\alpha }}=\int_{\tau_{1}}^{\tau_{N}}\frac{\gamma (\ln\tau )}{1+j\omega \tau }\mathrm{dln}\tau$$

where *γ*(ln*τ*) represents the distribution of each polarization process across timescales, which can be accurately approximated by summing RBFs centered at m characteristic times. The adopted time scale is typically logarithmic.

(3) $$\gamma \left(\ln\tau \right)=\sum_{m=1}^{M}x_{m}\phi_{\mu }\left(\left|\ln\tau -\ln\tau_{m}\right|\right)$$

where *x*_m_ is the weight coefficient of each RBF; *τ*_m_ and *µ* denote the center timescale and shape parameter of Gaussian RBF. M is the number of RBFs.

However, estimating these parameters from EIS data poses a challenge due to the ill‐posed problem of calculating the distribution function. Tikhonov regularization provides a generalized and effective solution, taking into account the quality of the measured noisy EIS data. The bi‐optimization objective function with a penalty term weighted by the second‐order regularization parameter *λ* can be written as follows^[^ ^21^ ^]^:

(4) $$\begin{array}{ccc} & & \arg\min\{\sum_{m=1}^{M}\left[{w^{\prime }}_{n}{\left(Z_{\mathrm{re}}^{\mathrm{sim}}\left(\omega_{n}\right)-Z_{\mathrm{re}}^{\exp}\left(x,\omega_{n}\right)\right)}^{2}\right.\\ & & \;\left.+{w^{\prime \prime }}_{n}{\left(Z_{\mathrm{im}}^{\mathrm{sim}}\left(\omega_{n}\right)-Z_{\mathrm{im}}^{\exp}\left(x,\omega_{n}\right)\right)}^{2}\right]\}+\lambda {\left\|L\cdot x\right\|}_{2}^{2} \end{array}$$

where x is the parameter vector to be estimated, including *x*_m_, *τ*_m_, and *µ* of each RBF. The length of the parameter vector is 3 m. *w′*_n_ and *w″*_n_ are the weight coefficients of two optimal objectives, which are 1 when only real and imaginary data are used for parameter identification.

As a result, the DRT curve deconvolved from EIS is a centralized representation of multiple RBFs on the timescale. The prominent DRT peaks indicate the presence of electrochemical reactions on the current time scale, with peak height reflecting the electrochemical reaction intensity. The integral over the single peak stands for the polarization resistance. Therefore, the quantification of polarization resistance is calculated by using the DRT peak area and total interfacial resistance.

(5) $$R_{p,i}=R_{\mathrm{int}}\cdot \frac{\int_{\tau_{l,i}}^{\tau_{u,i}}\gamma (\ln\tau )d\ln\tau }{\int_{-\infty }^{+\infty }\gamma (\ln\tau )d\ln\tau }$$

where *τ_u_* and *τ_l_* are the upper and lower boundary of the time‐scale of the *i*‐th peak. The peak area denotes a specific reaction's polarization resistance *R_p_* contribution to the total cell polarization.^[^ ^22^, ^24^ ^]^ Open‐source DRT Tools software based on MATLAB was adopted to calculate DRT from the reconstructed EIS.^[^ ^31^ ^]^ The Levenberg–Marquardt iteration method was utilized to identify the parameters of the Gaussian RBF, form the DRT plot, and then calculate the polarization resistance and time constants.

### 2.2 Three‐Electrode Battery Integrated Modeling

**Figure 2:**
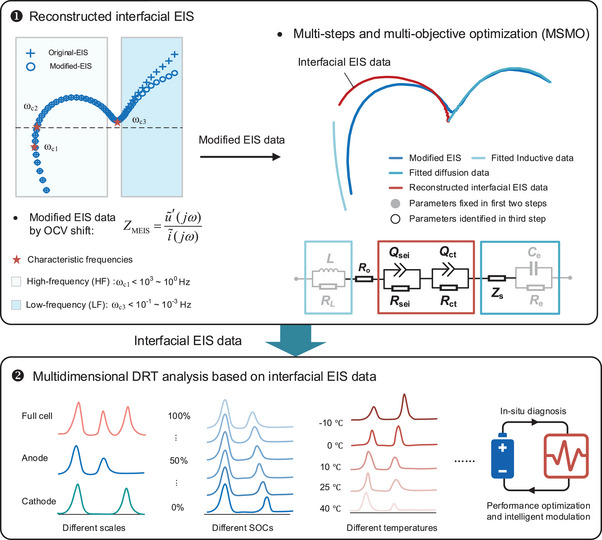
Schematic diagram of the proposed hierarchical analytical framework.

To elucidate crucial electrochemical processes with minimal computational complexity, we present an integrated model (IM) in this paper, as depicted in **Figure** **2**. This model comprises three components: 1) OCV reconstruction models to capture thermodynamic aspects; 2) ECMs to represent inductance and interfacial reaction processes; and 3) simplified electrochemical impedance models to describe solid‐ and liquid‐phase diffusion processes. The first and third components incorporate electrochemical phase transitions and reaction mechanisms. The latter two parts are collectively denoted as the impedance model representing the kinetic processes involved.

In the first part, to achieve the cell balancing between electrode and battery operational voltage windows, the multiple optimization objectives of minimizing the root‐mean‐square error (RMSE) between the measured open circuit voltage (OCV_mea_) and the simulated one (OCV_sim_) can be written as follows.

(6) $$FF_{V}=\sum_{j=1}^{3}\sqrt{\sum_{k=1}^{N_{t}}\frac{1}{N_{t}}{\left({\mathrm{OCV}}_{\mathrm{sim},k}^{j}-{\mathrm{OCV}}_{\mathrm{mea},k}^{j}\right)}^{2}}$$

where *N*_t_ is the number of OCV‐SOC data processed by the normalization of SOC. When *j* equals 1, 2, and 3, it represents the cathode, anode, and full cell. The OCV datasets of the electrodes come from the average 0.1 C charge and discharge data of half‐cell, while that of the full cell was from the rest period of the EIS test. To identify the thermodynamic parameters from the above OCV datasets, the following models simulated three‐electrode OCVs.

(7) $$\left\{\begin{array}{c} {\mathrm{OCV}}_{\mathrm{sim}}^{\mathrm{pe}}=f_{p}\left(1-\left(K_{p}\cdot SOC+S_{p}\right)\right)\\ {\mathrm{OCV}}_{\mathrm{sim}}^{\mathrm{ne}}=f_{n}\left(1-\left(K_{n}\cdot SOC+S_{n}\right)\right)\\ {\mathrm{OCV}}_{\mathrm{sim}}^{\mathrm{fc}}={\mathrm{OCV}}_{\mathrm{sim}}^{\mathrm{pe}}-{\mathrm{OCV}}_{\mathrm{sim}}^{\mathrm{ne}} \end{array}\right.$$

where *K* is the ratio of the electrode capacity to the full‐cell capacity Q; S is the offset of the OCV‐SOC curves of the electrode at full‐cell scale. The subscripts *p* and *n* are positive and negative electrodes. The RMSEs of cell balancing are less than 8 mV at each temperature.

In the impedance model, the cell impedance is the sum of the impedance of the two electrodes. The impedance of each electrode is further divided into the inductive process (l) and five kinetic processes: ohmic effect (ohm) related to electronic and ionic conductivity, interfacial reactions involving ions migration across SEI films (sei), and charge transfer (ct) occurring at the solid‐liquid interface, as well as diffusion process (d) within the electrolyte (e) and solid particles (s). Consequently, the cell impedance can be expressed as follows.

(8) $$Z_{c}=Z_{p}+Z_{n}=\sum_{k=1}^{2}Z_{l}^{k}+Z_{ohm}^{k}+Z_{sei}^{k}+Z_{ct}^{k}+Z_{e}^{k}+Z_{s}^{k}$$

where *Z_p_* and *Z_n_* are the impedance of positive and negative electrodes, respectively. The subscribe “k” takes values 1 and 2, denoting positive and negative electrodes. From left to right, the impedance model of each kinetic process is given in turn. The coupled inductive and interfacial impedance of individual electrodes is written below.

(9) $$\begin{array}{ccc} Z_{l}+Z_{int} & = & Z_{l}+Z_{sei}+Z_{ct}\\ & & =\frac{L}{1+j\omega \tau_{L}}+\frac{R_{sei}}{1+{\left(j\omega \tau_{\mathrm{sei}}\right)}^{\alpha_{sei}}}+\frac{R_{ct}}{1+{\left(j\omega \tau_{\mathrm{ct}}\right)}^{\alpha_{ct}}} \end{array}$$

where *L*, *R*, and *τ* are inductive, resistance, and time constant, respectively, int denotes interfacial impedance, including SEI and charge transfer processes.

Accounting for ion diffusion inside the electrolyte, we derived liquid‐phase diffusion impedance between the working electrode (WE) or counter electrode (CE) and reference electrode (RE) by introducing the RE concentration. The overpotential response arising from the difference in liquid phase concentration is represented as a first‐order R/Q network in Equation (10). Detailed derivation and validation have been provided in the supplementary file.

(10) $$Z_{e}\approx \frac{R_{e}}{1+j\omega \tau_{e}}$$

where *R_e_* and *τ_e_* are resistance and time constant; Both are lump parameters of the liquid‐phase diffusion process.

In the EIS test, diffusion‐driven solid‐phase concentration undergoes cyclic variation in response to external alternating current (AC) or voltage. By combining Fick's second law with the concentration boundary conditions and the average pore wall flux, the frequency‐domain response of the concentration difference on the active materials' surface and at its center to average AC current was described,^[^ ^34^ ^]^ as follows.

(11) $$\frac{{\tilde{c}}_{s}^{\mathrm{diff}}\left(\omega \right)}{\tilde{I}\left(\omega \right)}=\frac{\tau_{s}}{3}\frac{\tanh\left(\sqrt{\tau_{s}j\omega }\right)}{\sqrt{\tau_{s}j\omega }-\tanh\left(\sqrt{\tau_{s}j\omega }\right)}-\frac{1}{j\omega }$$

where *c_s_* and *τ_s_* stand for concentration and time constant of solid‐phase diffusion. The superscript “diff” denotes the difference between the surface and average concentration of the solid particles.

Substituting the variation of OCV induced by the altered concentration in Equations (11) into (12) results in the overpotential response arises from the difference in solid phased concentration:

(12) $$\begin{array}{ccc} Z_{s} & = & \left(\frac{\partial OCV}{\partial {\tilde{c}}_{s}^{\mathrm{diff}}}\right)\frac{{\tilde{c}}_{s}^{\mathrm{diff}}}{\tilde{I}}\\ & & =\frac{DV\cdot \tau_{s}}{3}\frac{\tanh\left({\left(\tau_{s}j\omega \right)}^{\alpha_{s}}\right)}{{\left(\tau_{s}j\omega \right)}^{\alpha_{s}}-\tanh\left({\left(\tau_{s}j\omega \right)}^{\alpha_{s}}\right)}-\frac{DV}{j\omega } \end{array}$$

where DV represents the differential OCV, calculated by reconstructed OCV in the hybrid model.

### 2.3 Hierarchical DRT Analytical Framework

To ensure accurate and reliable interfacial DRT, addressing issues of uncertainty in EIS data preprocessing and DRT analysis is crucial. Here, we propose a hierarchical enhanced analytical framework, as illustrated in Figure 2. This framework is divided into data processing and multi‐dimensional DRT. Data preprocessing as the first step includes modified EIS (MEIS) and reconstructed interfacial EIS (IEIS). The impedance is modified by OCV offset in the LF region to accurately reflect the true dynamics characteristics. Then, MEIS data is segmented into HF and LF data using characteristic resonance and transition frequencies for accurate impedance simulation. Leveraging the segmented data, an MSMO optimization is used to determine static parameters in inductive and diffusion models and then identify the interfacial model parameters, such as SEI and CT processes. Then, we can extract pure interfacial impedance by extending the impedance model across frequency, decoupling interfacial reactions from inductance and diffusion processes. In the second step, multi‐dimensional DRT analysis is performed using the pure interfacial impedance from the upper layer. It enables us to decouple interfacial kinetic processes by timescale identification and further analyzes the electrochemical mechanism of kinetic evolution under different SOCs and temperatures by timescale‐based characteristic factors. These results underscore the potential of the proposed method for in situ diagnostics on the evolution of electrochemical mechanisms under different stresses, thereby guiding battery performance optimization and intelligence modulation.

#### 2.3.1 Modified EIS by OCV Offset

In EIS tests, despite the small amplitude of the perturbation signals, the OCV change induced by the low‐frequency perturbation signal tends to be comparable to the voltage amplitude of the signal itself. This phenomenon compromises the ability of EIS in the low‐frequency region to accurately reflect the true kinetic characteristics of the battery. To address this issue and obtain EIS data that faithfully represents the genuine kinetic behavior, we adopt a modified method reported by Ruan et al.^[^ ^41^ ^]^ This method involves preprocessing the impedance data to mitigate the OCV shift caused by variations in current amplitude. Accordingly, we utilize DV obtained in cell balancing to quantify the extent of voltage shift induced by the alternating current. For the initial charging state SOC_0_, the AC capacity and voltage signals resulting from the AC voltage perturbation are as follows:

(13) $$\left\{\begin{array}{c} \tilde{q}=\int_{0}^{t}I\sin(\omega_{n}t-\varphi )dt=I/\omega_{n}\cdot \left[1-\cos(\omega_{n}t-\varphi )\right]\\ {\tilde{u}}^{\prime }=U\sin\left(\omega_{n}t\right)-f_{k}\left(\mathrm{SO}C_{0,k}+\tilde{q}/\omega_{n}Q_{\max,k}\right)+OCV_{k} \end{array}\right.$$

where *I* and *U* are the instantaneous values of alternating current $\tilde{i}$ and voltage signals $\tilde{u}$. *φ* denotes the phase angle. $\tilde{q}$ is the instantaneous capacity. *f* is the OCV‐SOC curves of an electrode or full cell.

From AC and modified voltage signals, the impedance reflecting the accurate dynamics information can be expressed as:

(14) $$Z_{\mathrm{MEIS}}=\frac{{\tilde{u}}^{\prime }\left(j\omega \right)}{\tilde{i}\left(j\omega \right)}$$

#### 2.3.2 Series Resonance from Cable Inductance and Interfacial Capacitance

To delve deeper into the hidden phenomenon of partial interfacial impedance induced by high‐frequency inductance, we employed an (RL)(RQ) model to simulate the evolution of characteristic frequencies relative to the proportion of inductance and capacitance. Notably, the interfacial impedance, encompassing the SEI film and charge transfer impedance, was represented by a first‐order R/Q network. For clarity, each parallel network can be expressed as follows.

(15) $$Z_{L}=\frac{R_{L}Lj\omega }{R_{L}+j\omega L}=\frac{R_{L}\omega^{2}L^{2}}{R_{L}^{2}+\omega^{2}L^{2}}+\frac{R_{L}^{2}\omega L}{R_{L}^{2}+\omega^{2}L^{2}}j$$

(16) $$Z_{R/Q}=\frac{R}{1+{\left(RQj\omega \right)}^{\alpha }}=\frac{R}{1+{\left(RQ\omega \right)}^{2\alpha }}-\frac{R{\left(RQ\omega \right)}^{\alpha }}{1+{\left(RQ\omega \right)}^{2\alpha }}j^{\alpha }$$

where *j* is the imaginary number.

According to Equations (15) and (16), the resonant circuit RLQ composed of (RL)(RQ) and ohmic resistance implies the cancellation of capacitive and inductive reactance, yielding the minimum real part of the impedance, denoted as *R*_min_. The resonant frequency *ω*_c1_ at which *R*_min_ occurs corresponds to the leftmost vertex in the EIS plot. According to fractional Euler's formula in Equation 17, we derived an equation of the impedance parameters and *ω*_c1_ at the resonance point.

(17) $$j^{\alpha }=e^{\frac{\alpha \pi }{2}j}=\cos\frac{\alpha \pi }{2}+j\sin\frac{\alpha \pi }{2}$$

(18) $$\frac{R_{L}^{2}L}{R_{L}^{2}+{\left(\omega L\right)}^{2}}j=\frac{R{\left(RQ\omega \right)}^{\alpha }}{1+{\left(R\omega L\right)}^{2\alpha }}\sin\frac{\alpha \pi }{2}j^{\alpha }$$

Directly solving the resonance frequency is challenging due to the fractional order present in the above equations. To address this, we interpolated the real part of the impedance and the corresponding frequency spectrum to identify the frequency at which the minimum resistance (Rmin) occurs. To determine the necessary of reconstruction by Nqusit plot or simple calculation, we can determine the necessity of the inductance for fitting the EIS data and the potential impact on interfacial DRT analysis by the relationship of *ω*_c1_ and the frequency intersecting the real part *ω*_c2_. The highest frequency of HF data for inductive impedance simulation is also segmented by characteristic frequencies *ω*_c1_. The highest frequency of 10^3^ Hz exceeds *ω*_c1_ to preserve partial coupled interfacial information.

#### 2.3.3 Multiple Step and Objective Optimizations of Model Parameters

To evaluate the quality of the impedance parameter sets during the global optimization process, fitness functions at minimizing the mean‐square error (MSE),^[^ ^42^ ^]^ the objective function for parameters identification can be constructed as:

(19) $$FF_{I}=\sum_{n=1}^{N}\left[\frac{1}{w}\cdot Re{\left(Z\left(\omega_{n}\right)\right)}^{2}+w\cdot Im{\left(Z\left(\omega_{n}\right)\right)}^{2}\right]$$

(20) $$Z\left(\omega_{n}\right)=\log\frac{Z_{\mathrm{sim}.}\left(\omega_{n}\right)}{Z_{\mathrm{MEIS}}\left(\omega_{n}\right)},n=1,2,\text{…},N$$

where *N* is the number of the impedance data points. Re and Im represent the real and imaginary parts of the impedance Z(*ω_n_*), respectively. *Z*_sim_ and *Z*_MEIS_ stand for the simulated and modified impedance of the LIBs. w is the weight factor, which is 0.5.

Nonetheless, optimization algorithms encounter challenges of local optima due to the excessive number of parameters requiring identification. We have adopted an MSMO approach to address this issue using the previously modified and segmented data. The primary objective of the MSMO approach is to reduce the model parameter count by pre‐identifying static parameters, that is, SOC‐independent parameters, such as inductive parameters (*R*_L_, *L*), from HF data (10^3^–10^0^ Hz) and electrolyte diffusion parameters (*R*_e_, *τ*_e_) from LF data (10^−1^–10^−2^ Hz). HF and LF data are segmented using characteristic frequencies *ω_c_*_1_ and *ω_c_*_3_. 10^3^ Hz exceeds *ω_c_*_1_ to retain partially coupled interfacial information, while 10^−1^ Hz is below *ω_c_*_3_ to separate interfacial and diffusion information. In the LF region, the goodness‐of‐fit (*R* ^2^) of the linear regression of the real and imaginary parts serves as an indicator to search for characteristic SOCs with highly identifiable liquid‐ and solid‐phase diffusion parameters, denoted as SOC_c_. The liquid‐phase diffusion parameters are identified by simulating LF data at SOC_c_ using an R(RC)Z_s_ model, which is considered constant across the entire SOC region. Here, 50% SOC_c_ was selected to fix liquid‐phase parameters. However, if linearity is high at all SOCs, the electrolyte‐related parameters will not be recognized, and only a single solid‐phase diffusion model can be used, particularly at low temperatures. According to the multi‐objective function in Equations (19) and (20), the model parameters of (RL)R(RQ) are estimated by fitting HF data with the inductive parameters assumed constant across the SOC region. The remaining parameters of (RL)R(RQ)(RQ)(RC)Z_s_ are identified at each SOC by presetting the recognized parameters (*R*_L_, *L*, *R*_e_, *τ*_e_). Consequently, we construct IEIS for DRT by extending the inductive and diffusion model over the overall frequency range, thereby decoupling the above two electrochemical and interfacial reactions from MEIS data. The expression is formulated as follows.

(21) $$Z_{\mathrm{IEIS}}\left(j\omega \right)=Z_{\mathrm{MEIS}}\left(j\omega \right)-Z_{s}\left(j\omega \right)-Z_{e}\left(j\omega \right)-Z_{L}\left(j\omega \right)$$

## 3 Results and Discussion

### 3.1 Analyzing Coupled Electrochemical Reactions by Numerical Simulations

From the theoretical derivations presented in the previous sections, we discovered a significant coupling between interface reactions and both the inductance and diffusion processes. The coupling necessitates numerical simulation to investigate the influence of the interconnected electrochemical reactions on the subsequent DRT analysis.

#### 3.1.1 Coupled Interfacial and Diffusion Impedance Analysis in Low‐Frequency Range

**Figure 3:**
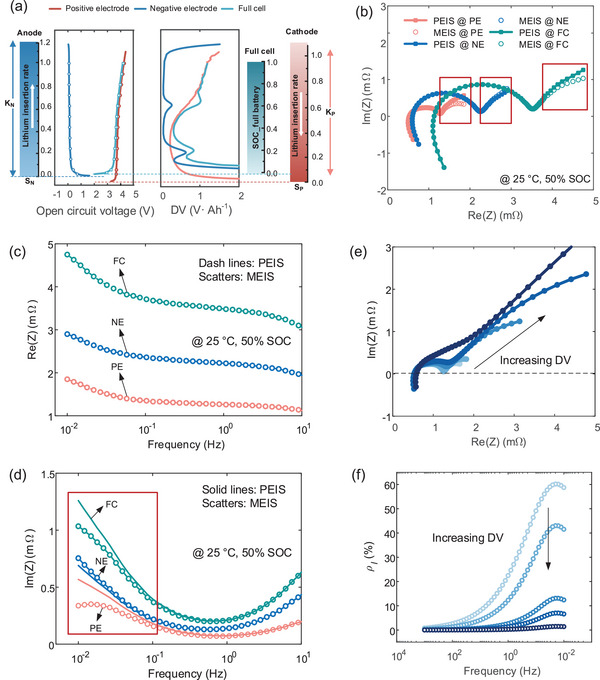
Coupled interfacial and diffusion impedance analysis. a) Matched OCV results along with the corresponding DV curve of three‐electrode batteries. b) PEIS and MEIS of three electrode batteries at 50% SOC and the associated c) real part and d) imaginary impedance. e) Variation of Nyquist plot with increasing DV values. f) Variation of *ρ_l_* over the frequency with the increased DV.

Owing to the almost invariant liquid‐phase diffusion impedance with SOC described in Equation 9, we investigate the need for modified EIS by examining DV values at different SOCs and scales. This allows us to discuss the potential impact of coupled interfacial and diffusion impedance on interfacial DRT analysis. **Figure** **3**a shows the OCV matching results of three electrode batteries and the corresponding DV values. As stated in Equation 13, the phase angle difference between the voltage offset signal caused by DV and the original voltage signal is π/2. In Figure 3b,c, we observe that the DV value affects only the imaginary part of low‐frequency potential EIS (LF‐PEIS) at 25 °C @ 50% SOC. The different shifted degrees and directions of LF‐MEIS in three‐electrode batteries can be observed by comparing PEIS and MEIS, attributing to inverse DV values of negative (NE) and positive electrode (PE) as well as a full cell (FC). Moreover, the PE (0.5 to 1.5 V·Ah^−1^) and FC (0.5 to 1.5 V·Ah^−1^) pose larger absolute DV values than the NE (0.1 to 0.5 V·Ah^−1^). This explains why more pronounced downward shifts occur in the LF impedance of PE and FC while a smaller upward shift is present in NE. Figure 3b displays that LF‐PEIS approximates a straight line, while LF‐MEIS appears semi‐circular. Previous literature has directly simulated LF‐PEIS with Warburge‐like elements, which ignores the electrochemical nature of diffusion processes and makes parameter identification of liquid‐phase diffusion difficult. MEIS showcases unexpected advantages in improving parameter recognizability at ambient temperatures.

To investigate the potential of MEIS in identifying solid and liquid‐phase diffusion parameters, numerical simulation was performed by varying DV on the anodic ECMs of 25 °C @ 50% SOC. The proportions of solid‐ and liquid‐phase diffusion are determined by DV. When DV is zero, there is no solid‐phase impedance in EIS, and all ionic impedance is determined by interfacial impedance. To illustrate the couple effect clearly, *ρ_l_* is introduced as the liquid‐phase fraction as a percentage of the total diffusion resistance. The baseline curve had a DV value of 1.15 V·Ah^−1^ and the proportion of electrolyte diffusion impedance (*ρ_l_*) of 12.68%, resulting in a distinct arc in LF‐EIS. The selected DV varies from 10^−2^ to 10^1^ V·Ah^−1,^ and the corresponding EIS trends with DV in Figure 3d. However, as illustrated in Figure 3e, increased DV value decreases *ρ_l_* over frequency, particularly in LF, making it difficult to distinguish the LF impedance. That is, the higher the linearity of LF‐MEIS, the lower *ρ_l_* and the more difficult it is to identify the electrolyte‐relevant parameters. Therefore, it calls for identifying electrolyte‐relevant parameters at a certain SOC point. Here, an interesting phenomenon is the liquid‐phase diffusion impedance of PE, which is more recognizable at low SOC, while the liquid‐phase diffusion impedance NE is at high SOC. This finding provides a direction for selecting SOCs to identify the electrolyte parameters. Additionally, we quantify the coupled degree of interfacial and diffusion processes, which provide a theoretical basis for the influencing factors of interfacial DRT in the next section.

#### 3.1.2 Coupled Interfacial and Inductive Impedance Analysis in the High‐Frequency Range

**Figure 4:**
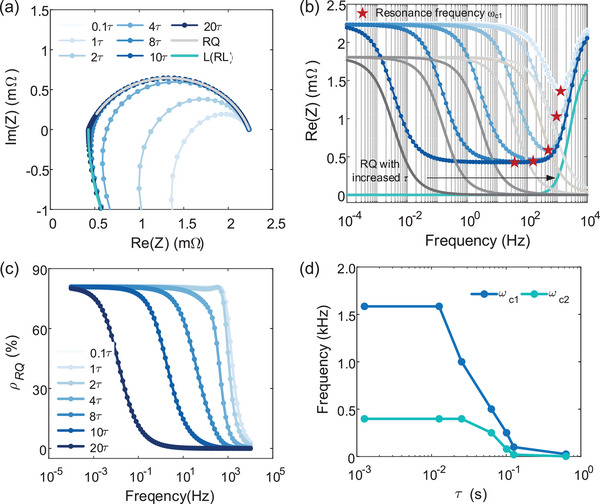
Coupled inductive and interfacial impedance analysis. a) Nyquist plot and b) real part of impedance with increased *τ*. *ρ*_RQ_ is the ratio of interfacial resistance in total resistance with increasing *τ* of interfacial reactions. c) Variation of *ρ*_RQ_ with increasing *τ*. d) The relationship between resonant and intersection frequency.

Due to the nearly unchanged current collector winding and coupled inductance with current‐dependent test cable,^[^ ^33^ ^]^ we investigate the variation of the Nyquist plot and the real part of impedance with the ratio of capacitive reactance in total resistance denoted as *ρ* in **Figure** **4** by adjusting the magnitude of interfacial reaction time constant *τ_int_* in numerical simulations. In Figure 4a, significant overlapping between the inductive and capacitive impedance is observed with decreasing *τ_int_*_,_ which is attributed to the cancellation of the imaginary part due to series resonance. The minimum real part of impedance, i.e., resonant impedance Re_min_, appears at *ω*_c1_ in Figure 4a,b. The evolution of characteristic parameters Re_min_ and *ω*_c1_ highly depends on the changed *ρ* due to *τ_int_*. As *τ_int_* increases, *ρ* shifts from a rapid to slow increase with decreasing frequency until it reaches a maximum value, as shown in Figure 4c. Meanwhile, *ω*_c1_ gradually decreases and approaches with *ω*_c2_ in Figure 4d, and Re_min_ decreases until a stable value, i.e., ohmic resistance. In Figure 4a, we observe that single inductance and capacitance models overlap entirely with the coupled EIS curves when *τ_int_* increases to 10 *τ_int_*, which suggests completely decoupled inductance and capacitance, resulting in *ω*_c1_ being equal to *ω*_c2_. Thus, the typical processing of discarding the portion below the real axis also eliminates part of the interfacial kinetic information containing SEI film and charge transfer processes. These results reveal the risk of inaccurate DRT results caused by the coupled inductance and interface processes and the necessity of the additional inductive model for high‐fidelity interfacial DRT analysis.

### 3.2 Influence of Coupled Electrochemical Reactions on Interfacial DRTs

**Figure 5:**
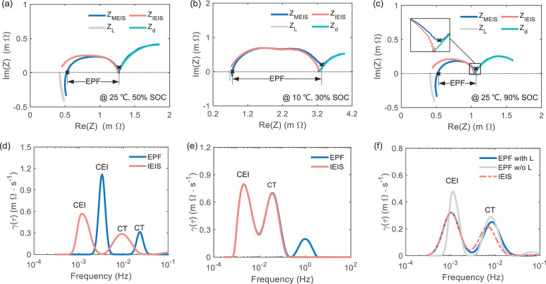
Influence of coupled electrochemical reactions on interfacial DRTs. Nyquist plots and corresponding DRT plots from MEIS and IEIS under different conditions. a,d) @ 25 °C, 50% SOC, b,e) @ 10 °C, 30% SOC, c,f) @ 25 °C, 90% SOC of cathode.

Given the observed severe couple effects of electrochemical processes in the numerical simulations, we hypothesized that the discrepancy in the interfacial DRT results could be attributed to the coupling of HF‐inductive and LF‐diffusive processes with interfacial reactions. To validate this hypothesis and enhance the precision of the DRT, we investigated the impact of coupled electrochemical reactions on interface DRT by comparing the results obtained through two data processing methods with the time constant determined in the HM in **Table** **1**. A common approach involves directly extracting partial frequency data from *ω_c_*_2_ and *ω_c_*_3_, referred to as EPF. As shown by two black crosses in **Figure** **5**a, *ω_c_*_2_ is the frequency at the intersection of impedance and *x*‐axis, and ω*_c_*_3_ is the frequency at a minimal imaginary absolute value above the *x*‐axis. Another method entails reconstructing pure interfacial impedance by discarding non‐interfacial impedance from extending the hybrid model across the frequency range, termed IEIS. Figure 5 illustrates the Nyquist plot and associated DRT results in three scenarios. Additionally, the hyperparameters of the DRT are set to a regularization order of 2, a regularization parameter of 1e‐4, and a shape factor of 0.5.

**Table 1:** Comparison of DRT results from EPF and IEIS with identified time constants in IM.

EIS conditions	DV [mV]	τ*_x_*	τ_EPF_ [s]	τ_IEIS_ [s]	τ_IM_ [s]	RE [τ_EPF_‐τ_IM_] [%]	RE[τ_IEIS_‐τ_IM_] [%]
@ 25 °C, 50% SOC	4.72	τ_cei_	3.31e‐3	1.19e‐3	1.09e‐3	−16.28	−1.27
		τ_ct_	2.21e‐2	8.93e‐3	8.64e‐3	−19.77	−0.70
@ 10 °C, 30% SOC	2.35	τ_cei_	2.09e‐3	2.07e‐3	3.02e‐3	6.34	6.51
		τ_ct_	3.58e‐2	3.67e‐2	3.87e‐2	2.40	1.63
@ 25 °C, 90% SOC	10.78	τ_cei_	1.03e‐3	1.03e‐3	1.00e‐3	−0.43	−0.43
		τ_ct_	6.86e‐3	8.79e‐3	7.77e‐3	2.56	−2.54

For the Nyquist plot @ 25 °C, 50% SOC depicted in Figure 5a, we observed severe coupling between inductive and interfacial impedance and minimal coupling between interfacial and diffusive impedance. This was attributed to the series resonance effect and lower DV values. Figure 5b indicated weak coupling between inductive and interfacial processes at 30% SOC of 10 °C, while at 90% SOC of 25 °C with a DV value of 10.78, strong coupling between diffusive and interfacial impedance was observed in Figure 5c. Two DRT peaks within the timescale of 10–4 to 10–1 were identified as cathode electrolyte interphase (CEI) film and CT processes for the cathode. Comparative DRT results and the corresponding DV values at the current SOC are listed in Table 1. Hidden interfacial impedance was revealed when compared with IEIS, as indicated by the pink and blue lines in Figure 5a, resulting in a pronounced rightward shift of two DRT peaks in Figure 5d. Furthermore, DRT_IEIS_ closely matched the identified time constants in IM than DRT_EPF_. These findings suggest that the coupled inductive and interfacial processes contribute to inaccurate DRT and biased simulations in the HF range in Figure S2 (Supporting Information). At 30% SOC of 10 °C, an additional DRT peak was observed in Figure 5e, explained by a pseudo‐peak at the end of the transition period from CT to diffusion processes. The unsusceptible interfacial DRT was an interesting phenomenon attributed to faint series resonance effects in the HF region and separable CT and diffusion processes due to DV close to zero in the LF region. It is evident that their detachability highly depends on DV. For 90% SOC of 25 °C with a high DV, more serious CT and diffusion overlap led to residual partial diffusion information in the EPF, as described in the zoom figure of Figure 5c. To illustrate DV‐induced DRT variation occurring at the CT process, when discussing the diffusive effect on the interfacial DRT, the fitted IM model excludes the inductance, denoted as EPF w/o L. At the same time, EPF was renamed EPF with *L* for clear distinction. As shown in Figure 5f, the inductance impacts the location and height of DRT_CEI_ and the remaining impedance in EPF w/o L results in a rightward shift of the DRT_CT_. The unaltered DRT_CEI_ further indicates that residual diffusive impedance only affects DRT_CT_ while not influencing DRT_CEI_. Notably, the implementation of reconstructed IEIS is rational and effective in suppressing the pseudo peaks and enhancing precise timescale identification using DRT.

### 3.3 Identifying Interfacial Kinetic Processes Using DRT Analysis

In Nyquist plots of EIS data, one or two semi‐circles appear in the middle‐frequency region, indicating complex interfacial processes. These processes encompass both static and dynamic processes. For instance, static processes such as contact resistance (CR) between particles and particle/current collector and ionic conduction in CEI and SEI remain relatively stable during charging or discharging. On the contrary, lithium concentration affects dynamic processes like interfacial CT, resulting in varied charge transfer impedance with SOC. The charge transfer processes of negative and positive electrodes are called CT‐NE and CT‐PE, respectively. In addition, these electrochemical processes also exhibit distinct sensitivities to temperature due to differences in active reaction energies. The reversibility of the kinetic processes concerning the SOC, alongside their correlative difference in temperature, effectively distinguishes between static (irreversible) and dynamic (reversible) processes. This distinction gives physical meanings to the observed DRT peaks and allows tracking the kinetic evolution of internal mechanisms that are affected by external factors.

**Figure 6:**
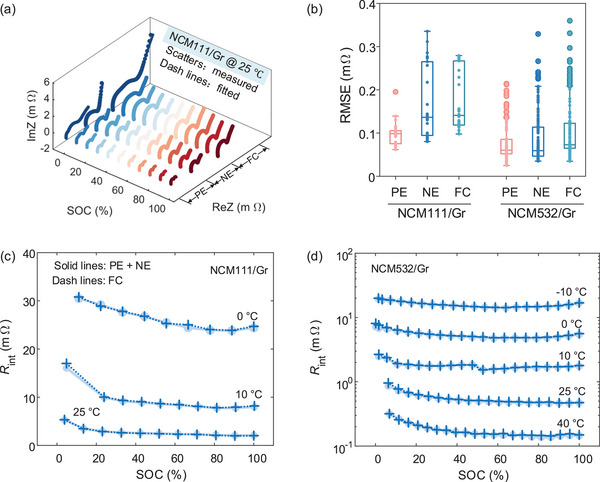
EIS fitting results of three‐electrode batteries and interfacial impedance relationship between electrode and cell. a) EIS fitting results for NCM111/Gr three electrode battery at 25 °C. b) Box plot of fitted RMSE for two types of three‐electrode batteries on the negative electrode, a positive electrode, and full cell. Through identified IM, interfacial impedance relationship between electrode and battery at various temperatures for c) NCM111/Gr and NCM532/Gr cells.

However, to further explore the identified timescale, correlation, and corresponding relationship of cross‐scale DRTs at different SOCs and temperatures, it is imperative to guarantee the consistency of interfacial impedance of cell (macro) with the sum of two electrodes (micro). We verified the relationship between interfacial impedance at micro and macro scales using the high‐fidelity IM, as depicted in **Figure** **6**. In Figure 6a, the simulated EIS closely aligns with the modified EIS, indicating the effective capture of the interfacial kinetic processes by the IM. The RMSE of fitting IM for two three‐electrode batteries is presented in Figure 6b, underscoring the high accuracy of the proposed model under varying conditions. Furthermore, we compared the sum of electrode interfacial resistances (R_int_) with the R_int_ of the cell in Figure 6c,d. The identical R_int_ results suggest that the proposed IM successfully decouples interfacial reactions with inductive and diffusive processes without sacrificing accuracy. That is, IEIS at the cell level encompasses all electrode interfacial information. The proposed data processing and comparison results are crucial for subsequent cross‐scale and multivariate DRT analysis and for ensuring the accuracy of DRT results.

#### 3.3.1 Quantifying Complex Kinetic Processes by SOC‐DRTs

**Figure 7:**
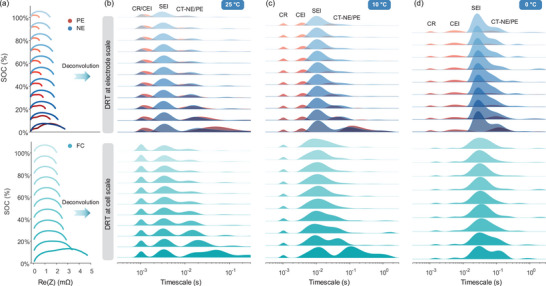
Multidimensional‐DRT analysis for NCM111/Gr three‐electrode battery. a) IEIS plots at electrode and cell scales at 25 °C. The corresponding relationship of DRT peaks between electrode and cell at b) 25 °C, c) 10 °C, and d) 0 °C. The first row represents the results at the electrode level, and the second denotes the results at the cell scale.

**Figure** **7**b shows the DRT plot deconvoluted from reconstructed IEIS data in Figure 7a, featuring some local maxima, each representing the contribution of the electrochemical processes to the total polarization. The position and area of the DRT peak over the logarithmic frequency coordinate identifies the time constants and polarization resistance. The change in the DRT profiles directly indicates the electrochemical nature and varied intensity of the electrode reactions. To display the changed separability of anodic and cathodic CT processes with SOC, we plot EIS (Figure 7a) and DRT profiles (Figure 7b–d) of two electrodes in the same subfigure. On the bottom of these subfigures, DRT plots of the cell possess the same timescale as the electrode to clarify the correspondence of DRT peaks between macro and micro scales.

According to the reversibility of these electrochemical reactions, the DRT peaks were marked as CR, CEI, and CT‐PE for the cathode and CR, SEI, and CT‐NE for the anode. However, the number of the DRT peaks at the cell level is always less than 6 due to the coupled effect of several kinetic processes with similar time constants, such as coupled CR and CEI at 25 °C, CEI and SEI at 10 °C, as well as coupled CT‐NE and CT‐PE. To showcase the correspondence of DRT peaks between the cell and electrodes, the assignment of DRT peaks, the corresponding timescales, and coupled behavior were listed in **Table** **2** for NCM111/Gr cells. In Table 2, CT without parentheses represents the distribution of time constants in the SOC region where CT_NE and CT_PE can be decoupled from the DRT plot of the full cell. In Figure 7b–d, we observed that the CR, CEI, and SEI with SOC are unaltered at all temperatures due to the SOC independence of the static processes. In contrast, the dynamic processes, like CT‐NE and CT‐PE, gradually shifted from coupled to separable at the cell scale from 100 to 0% SOC at 25 °C. The timescale of cathodic CT changes from 1e‐2 – 2e‐2 to 5e‐2 s at less than 5% SOC. This phenomenon is explained by the fact that the time constants of cathodic CT vary more significantly than anode at low SOC. Although this trend can still be found at other temperatures, CT‐NE and CT‐PE cannot be distinguished due to their similar timescales, e.g., 6e‐2 – 2e‐1 s at 10 °C (Figure 7c) and 6e‐2 – 2e‐1 s at 0 °C (Figure 7d). Moreover, SEI and CEI were coupled at 10 °C due to the close time constants of 9e‐3 s and 3e‐3 s, whereas the disparity in time constants enables the complete separation of CEI and SEI at 0 °C.

**Table 2:** Correspondence of kinetic processes at the cell and electrode scales and their time constants for NCM111/Gr cells.

T [°C]	Peak No.	Full cell		Negative electrode		Positive electrode	
		Kinetic	*τ* (s)	Kinetic	*τ* (s)	Kinetic	*τ* (s)
25	1	(CR|CEI)	1e‐3	CR	1e‐3	(CR|CEI)	1e‐3
	2	SEI	3e‐3	SEI	3e‐3	−	−
	3	(CT)	1e‐2 – 2e‐2	CT	1e‐2 – 2e‐2	CT	1e‐2 – 2e‐2
	4	CT	5e‐2	−	−	CT	5e‐2
10	1	(CR|CEI)	1e‐3	−	−	CR	1e‐3
	2	(CEI|SEI)	9e‐3	SEI	9e‐3	CEI	3e‐3
	3	(CT)	6e‐2 – 2e‐1	CT	6e‐2 – 1e‐1	CT	3e‐2 – 2e‐1
0	1	CR	1e‐3	−	−	CR	1e‐3
	2	CEI	4e‐3	−	−	CEI	5e‐3
	3	SEI	3e‐2	SEI	3e‐2	−	−
	4	(CT)	6e‐2 – 2e‐1	CT	9e‐2 – 1e‐1	CT	6e‐2 – 2e‐1

^a)^ Peak represents the number of left‐to‐right DRT peaks at the cell scale;

^b)^ the associated electrode kinetic processes are listed in the corresponding rows, and () implies several coupled kinetic processes at the current peak position;

^c)^ (CT) denotes coupled charge transfer processes of the anode and cathode;

^d)^ The timescales are merely for the reader's convenience. Separable time constant plots were tallied with more precise results.

**Figure 8:**
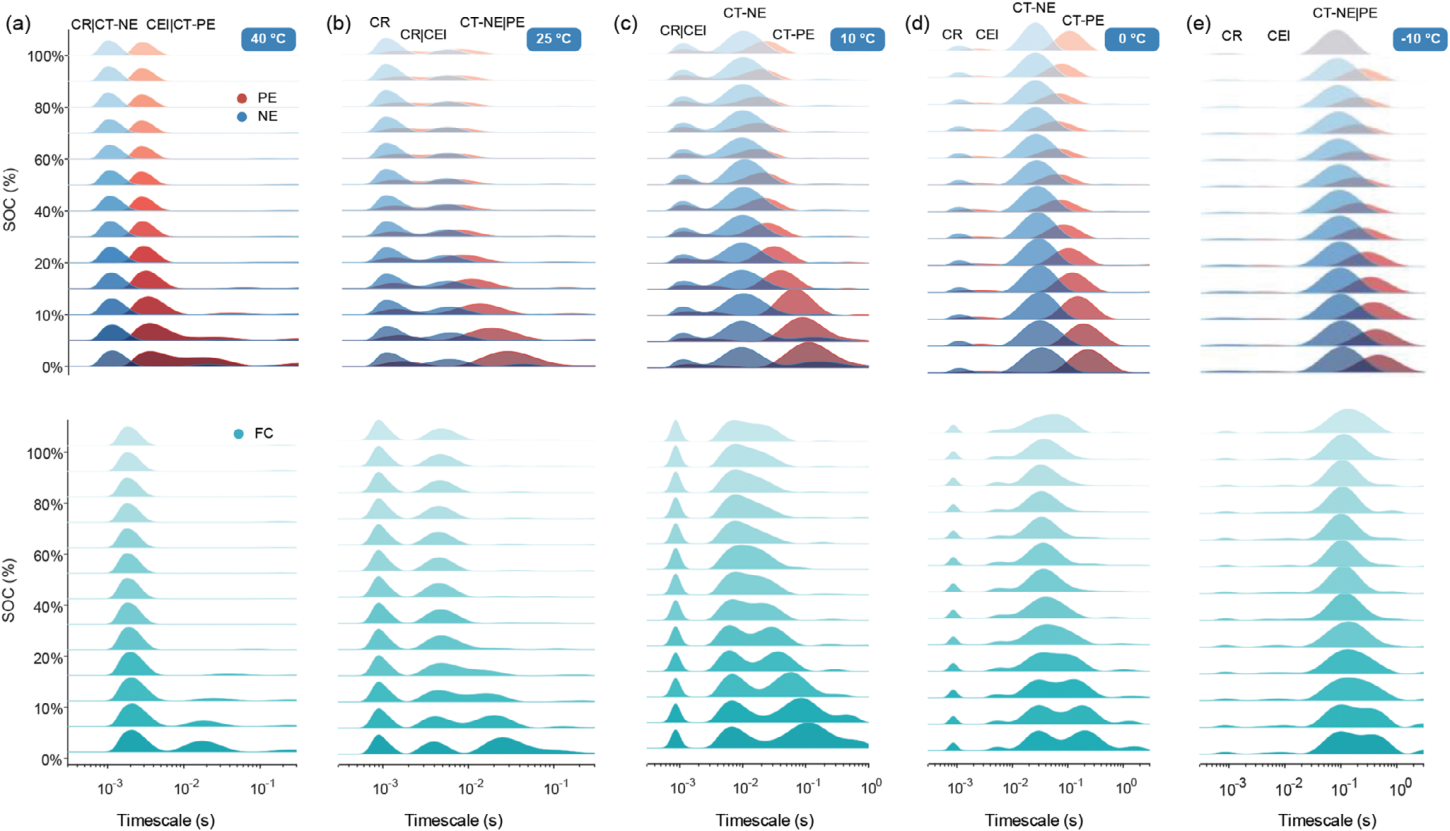
Multi‐dimensional DRT analysis for NCM532/Gr three‐electrode battery. The corresponding relationship of DRT results for NCM532/Gr three‐electrode battery at a) 40 °C, b) 25 °C, c) 10 °C, d) 0 °C, e) −10 °C. To save space, only DRT results are displayed at specific SOCs.

**Figure 9:**
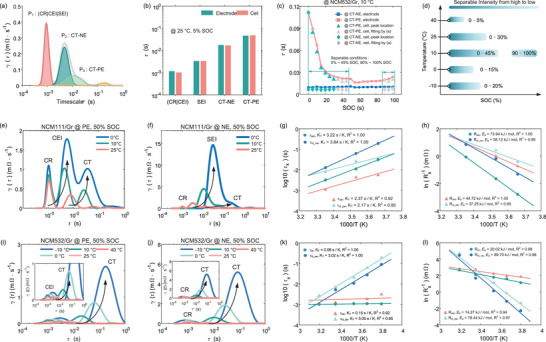
The validation of separable timescale and temperature‐dependence analysis of kinetic processes. a) The peak fitting results of the DRT plots of NCM532/Gr cells at 25% SOC of 25 °C. The validation results of identified time constants between cell and electrode level for b) NCM111/Gr at 5% SOC of 25 °C and c) NCM532/Gr cells at 10 °C. d) The separable condition of NCM532/Gr cells is counted at various temperatures and SOCs. DRT evolution of e,i) cathodic and f,j) anodic interfacial kinetic processes at various temperatures. Linear fitting relationship of *τ_x_* as a function of T g) NCM111/Gr and k) NCM532/Gr cells. Arrhenius plots in the format of ln(R*_x_* ^−1^) as a function of 1000/T for h) NCM111/Gr and i) NCM532/Gr cells.

Similarly, the DRT plots and timescale distribution of NCM532/Gr three‐electrode battery were displayed in **Figure** **8** and **Table** **3**, respectively. Surprisingly, there is no observed response peak of SEI at all temperatures in the DRT results, arising from the insignificant electrochemical properties due to the very thin SEI membrane. Another piece of evidence is that the time constants of SEI (1e‐3 – 1.6e‐2 s) generally exceed that of CR (1.6e‐6 – 3e‐6 s) and CEI (1.6e‐4 – 6.7e‐3 s) according to the review work from Lu et al. The absence of SEI DRT peaks further supports a trivial electrochemical process resulting from the extremely thin SEI film. Thus, there are only static CR and dynamic CT processes for the negative electrodes of the NCM532/Gr battery. An interesting phenomenon is that the time constant of cathodic CT gradually deviates from the anodic CT with decreased SOC at all temperatures. This enables the separation of CT‐PE and CT‐NE at the cell level in Figure 8a–e. In Figure 8a, the time constants of cathodic CT can be separated from the anode at 5% SOC even if kinetic processes heavily overlap at 40 °C. In this case, CT‐PE exhibits an evolution range from 3e‐3 s to 2e‐2 s, whereas the anodic evolution of CT‐NE is located at 1e‐3 s and coupled with CR and CEI. In addition, the asymmetric DRT peak may contain coupled kinetic processes of CT‐NE and CT‐PE, such as the middle SOC region at 10 °C (Figure 8c) and the low SOC region at −10 °C (Figure 8e). To isolate CT‐NE and CT‐PE within the asymmetric DRT plot, we fitted the DRT plot using the Gaussian function to estimate the time constants of the CT‐PE peak, as shown in **Figure** **9**a. Nonetheless, time constants of separable peaks can be directly identified by peak location, like 5% SOC of 25 °C for NCM111/Gr cells. In Figure 9b, the separable results almost align with that at the electrode scale, proving the effectiveness of timescale identification at 5% SOC on non‐invasive decoupling kinetics. For NCM532/Gr cells, the identified time constants by peak location and fitting were marked as green and grey scatters in Figure 9c. Compared with the identified results at the electrode level, we discovered that the DRT results of the cell at 10 °C can effectively reproduce the electrode kinetic information. In Figure 9d, we further counted all separable conditions at various temperatures to provide theoretical guidance for the non‐invasive diagnosis of polarization degradation. The low SOC region at ambient temperature for NCM111/Gr battery and all temperatures for NCM532/Gr battery are favorable conditions, enabling non‐destructive separation of electrode's interfacial reactions at the cell scale. The excellent separable presentation in low SOC conditions can be explained by the phase‐dominated effect of cathode materials in higher oxidation states (such as Mn^4+^), highlighting the proposed method's potential advantages on non‐destructive diagnosis.

**Table 3:** Correspondence of kinetic processes at the cell and electrode scales and their time constants for NCM532/Gr cell.

T [°C]	Peak	Full cell		Negative electrode		Positive electrode		
		Kinetic	*τ* (s)	Kinetic	*τ* (s)	Kinetic	*τ* (s)	
40	1	(CR|CEI| CT)	2e‐3	(CR|CT)	1e‐3	(CR|CEI| CT)	3e‐3	
	2	CT	2e‐2	−	−	CT	2e‐2	
25	1	(CR|CEI)	1e‐3	CR	1e‐3	(CR|CEI)	1e‐3	
	2	(CT)	5e‐3	CT	6e‐3	CT	7e‐3	
	3	CT	2e‐2 – 3e‐2	−	−	CT	3e‐2	
10	1	(CR|CEI)	9e‐4	CR	1e‐3	(CR|CEI)	1e‐3	
	2	(CT)	6e‐3	CT	1e‐2	CT	1e‐2	
	3	CT	2e‐2 – 8e‐2	−	−	CT	2e‐2 – 8e‐2	
0	1	CR	9e‐4	CR	1e‐3	−	−	
	2	CEI	5e‐3	−	−	CEI	3e‐3	
	3	(CT)	4e‐2	CT	2e‐2	CT	7e‐2 – 1e‐1	
	4	CT	9e‐2 – 3e‐2	−	−	CT	1e‐1 – 3e‐1	
−10	1	CR	9e‐4	CR	9e‐4	−	−	
	2	CEI	4e‐3	−	−	CEI	4e‐3	
	3	CT	8e‐2	CT	8e‐2	−	−	−
	4	CT	4e‐1	−	−	CT	4e‐1	

#### 3.3.2 Quantifying Temperature Dependence of Complex Kinetic Processes by Temp‐DRTs

To reveal the temperature dependence of each electrochemical reaction by quantifying the characteristic parameters under low temperatures, such as timescales and resistance, interfacial DRT data of electrodes at each temperature was collected. As described in Figure 9e,f, we observed a significant change in the height and location of DRT peaks for NCM111/Gr cells, indicating the retarded timescale and enhanced resistance on the reaction interface with decreased temperatures, like CEI, SEI, CT‐PE, and CT‐NE. However, different degrees of change in DRT peaks govern which reactions are the control steps for LT performance. The sharply increased peak height of SEI and the significant right‐shifted peak location of CT suggest the dominant effects of SEI and CT‐NE at low temperatures. As shown in Figure 9g,h, the change rate of timescales (*K*_T_) and activation energy (*E*_a_) of each electrochemical reaction can be deduced from the R/τ‐T relationship, demonstrating the LT predominate kinetics of SEI and CT‐NE from ranking K_T_ and *E*_a_ values. The activation energy of CEI is 14.27 and 44.72 kJ mol^−1^, which is almost consistent with the range of activation barrier of SEI, 19.2 to 69 kJ mol^−1^, obtained by Illig et al.^[^ ^43^ ^]^ Although the activation energy of SEI is 73.94 kJ mol^−1^ within NCM111/Gr cells exceeds the range of 53–60 kJ mol^−1^, it still is in the same order of magnitude as our results, which points to SEI as the dominant factor limiting LT performance. CT‐PE and NE activation energy for NCM111/Gr cells is 37.25 and 58.12 kJ mol^−1^, close to the 52–61.7 kJ mol^−1^ range counted by Zhu et al.^[^ ^32^ ^]^ NCM532/Gr cells have higher *E*_a_ values of CT‐PE (89.70 kJ mol^−1^) and CT‐NE (78.44 kJ mol^−1^) than NCM111/Gr cells. Surprisingly, we observed a remarkable increase in the peak height of CT‐NE in NCM532/Gr cells rather than SEI, which was distinguished from NCM111/Gr cells. Since the SEI peak has a tiny weight, parameters related to SEI can be overlooked when identifying polarization processes. Only anodic *R*_cr_ and *R*_ct_ne_, along with cathodic *R*_cei_ and *R*_ct_pe_, influence the LT critical kinetics shown in Figure 9i,j. Moreover, the sluggish charge transfer kinetics are identified as the chief culprit by higher *K*_T_ and *E*_a_ values than other DRT results from Figure 9k,l, which also supports the rapid increase of peak height of CT. It indicates that the anodic charge transfer process becomes the rate‐limiting process at low temperatures. Consequently, the temperature dependence of polarization resistances, such as SEI and CT, should be responsible for the abrupt change of impedance and capacity. Notably, substituting electrolyte composition and concentration and the specific properties of electrode materials, such as manufacturing processes, particle size, morphology, and surface treatments, also result in different changes in activation energy. The LT control steps should be cautiously analyzed using the proposed method. Therefore, the LT performance of LIBs can be improved by the modulation of the rate‐limiting process, such as optimizing the electrolyte and electrode materials, surface modification, applying pressure, etc.

### 3.4 Limitations and Outlook

In this work, our focus is on the cross‐scale decoupling of kinetic processes in interfacial electrochemical reactions. We do not delve into diffusion kinetic processes due to several reasons. First, the low‐frequency impedance model is not compatible with the DRT theory. Additionally, the characteristic parameters are difficult to identify due to the strong coupling effects between solid‐ and liquid‐phase diffusion, especially under low temperature and low SOC conditions. In subsequent investigations, our primary objective is to expand upon decoupling diffusion kinetics. This will involve conducting global sensitivity analyses of electrochemical parameters and identifying these parameters through longer timescale experiments, such as continuous charging and discharging processes, along with ultra‐low frequency EIS. Although experiment‐based and data‐driven identification methods of diffusion processes have been previously developed to decouple impedance and polarization voltage of liquid and solid‐phase diffusion, few scholars have implemented sufficient validation of the proposed decouple methods. Three‐electrode batteries should be widely extended in subsequent studies as a conducive means of adequate validation of decoupling kinetic processes.

In the future, we aim to expand the application scope of this work in new electrochemical systems and wider dimensions. The developed non‐invasive kinetic analytical framework will be further adapted for new electrochemical systems on diverse experimental conditions, such as the aging state, mechanical stress, vibration amplitude, lithium plating degree, etc. Such a large‐scale experiment matrix achieves the wider dimensions analysis to track stress‐driven kinetic evolution for deep insight into cell chemistry. Kinetic evolution further guides the identification of physical‐driven parameters for refined multiphysics simulation. Another extent is to establish a correlation between internal microscopy mechanisms and external macroscopy performance through DRT characteristic factors, thus facilitating the modulation of operation conditions and the safety boundary of batteries.

## 4 Conclusion

This work aimed to create a hierarchical analytical framework suitable for low interfacial impedance batteries to cross‐scale decouple kinetic processes, to be associated with future non‐destructive degradation diagnosis with the capability to take advantage of the multi‐dimensional DRT technique in timescale identification. This framework incorporates data processing to reconstruct interfacial impedance for enhancing DRT resolution and multi‐dimensional DRT analysis to validate kinetic timescale for reducing the uncertainty of DRT. Leveraging an integrated frequency‐domain model combining thermodynamic and kinetic behavior, pure interfacial impedance was reconstructed for the first time by eliminating the simulated inductive and diffusive impedances across frequencies. The simulation results indicated that the coupled inductance and diffusion processes affect the identifiability of the interfacial DRT, thus contributing to inaccurate timescale identification. A comparison of DRT results with different data preprocessing demonstrated that these coupled processes led to the shifted peak height and location of the interfacial DRT, showcasing the importance of practical data preprocessing for improving timescale identification by ≈20%. Multi‐dimensional DRT decoupled the static and dynamic kinetics at multi‐scales by the reversibility of SOC, thus verifying the consistency of timescales identified across electrode and cell scales. The identified timescales bridge the correlation of various kinetic processes between electrode and cell scales. The cross‐scale DRT analysis decoupled kinetic processes by timescale identification, thus revealing that timescale at low‐SOCs of 25 °C can separate electrode interfacial polarization from cell level due to a more significant change in cathodic charge transfer with SOC than the anode. The characteristic indicators, such as identified timescales and polarization resistances, revealed different control steps for low‐temperature performance within different batteries, such as LT performance controlled by anodic solid electrolyte interphase in NCM111/Gr cell or charge transfer in NCM532/Gr cell. The software encapsulating the proposed analytical method was open‐access on the website (https://github.com/xuecaii/Multi‐DRT.github.io/releases/tag/v1.0.0) to enable one‐shot non‐invasive diagnostics for complicated kinetic degradation.

## 5 Experimental Section

### Test Batteries and Experimental Platform

This work adopted two types of commercial pouch cells with low IEIS. The high‐power cells use LiNi_0.3_Co_0.3_Mn_0.3_O_2_ (NCM111) as cathode materials and graphite (Gr) as anode materials, referenced as NCM111/Gr cells. The high‐energy cells utilized LiNi_0.5_Co_0.3_Mn_0.2_O_2_ (NCM532) and Gr as electrode materials, referred to as NCM532/Gr cells. Detailed specification parameters of the cells are listed in **Table** **4**. Low interfacial impedance poses significant difficulties for decoupling kinetic processes due to the severely coupled electrochemical reactions. To further explore the effectiveness of the proposed framework on timescale identification, EIS measurements were conducted on the self‐made three‐electrode batteries under different conditions. A Bio‐Logic electrochemical station was used for EIS tests of three‐electrode batteries, and a temperature chamber was used to control the battery temperature. A T‐type thermocouple was attached to the surface of the battery, and the temperature data was collected using the HIOKI data acquisition instrument.

**Table 4:** Specifications of the lithium‐ion battery used in this work.

Item	NCM111/Gr cells	NCM532/Gr cells
Type	High‐power	High‐energy
Cathode material	LiNi_0.3_Co_0.3_Mn_0.3_O_2_	LiNi_0.5_Co_0.3_Mn_0.2_O
Anode material	Graphite	Graphite
Normal capacity (Ah)	8	55
Charge cut‐off voltage (V)	4.2	4.2
Discharge cut‐off voltages (V)	2.75	2.5
Charge current rate (C)	8	1
Discharge current rate (C)	10	2
Temperature range (°C)	−5 – 40	−10 – 40

### Three‐Electrode Battery Preparation

**Figure 10:**
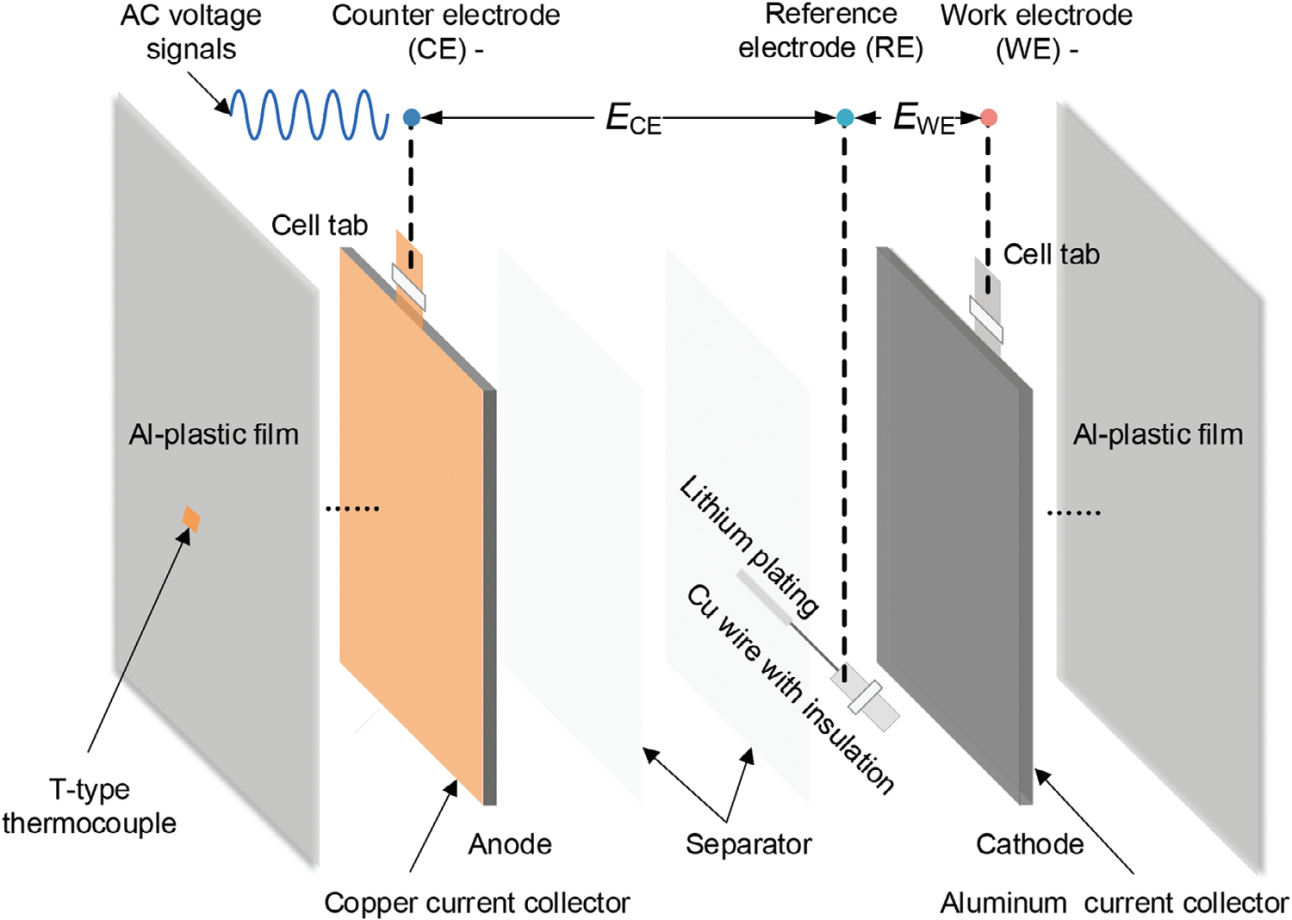
Schematic illustration of the refitted commercial three‐electrode batteries.

**Figure 11:**
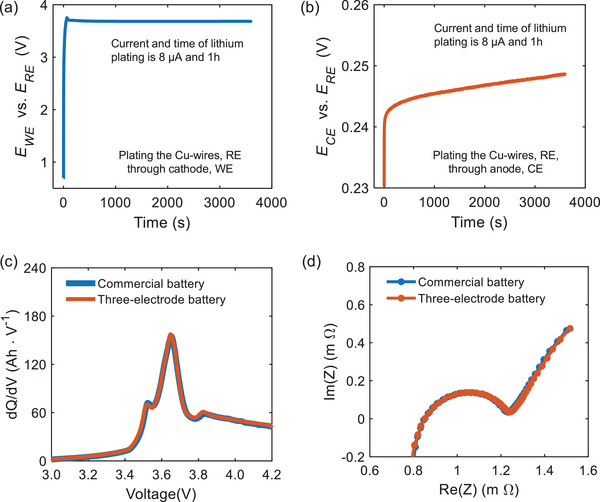
Potential variation of a) work and b) counter electrode in plating to the Cu‐wires that is a reference electrode. c) Incremental capacity curve and d) EIS at 50% SOC of three‐electrode and commercial battery.

The three‐electrode batteries were fabricated by inserting the reference electrode into the multi‐layered commercial pouch cells (**Figure** **10**). The battery was fully discharged at 0.1 C to ensure experiment safety before fabricating the three‐electrode batteries. The acquirement and implementation of the reference electrode were completed in a glovebox filled with argon gas. First, the 20 µm diameter Cu‐wire was immersed in dilute sulfuric acid at both ends and then rinsed with ethanol to remove surface insulation and impurities. The dipped length of the Cu‐wire was 2 cm. One end of the Cu‐wire was inserted into the battery for subsequent lithium plating (LP), and the other was soldered to the reference electrode lugs to install the measuring clamps. Next, the aluminum foil was opened. Then, part of the electrode rolls was unfolded, followed by the additional separator placed between the Cu‐wire and electrode to avoid the internal short circuit. Then, the exposed end of the Cu‐wire was placed between the separators. Finally, the inner core was restacked with a new Al‐plastic film, and a small amount of electrolyte was injected through the filled port. The manufactured three‐electrode cell was evacuated after resting for more than 24 h. The LP sequence was used to plate the Cu‐wires through the cathode, and then the anode deposits uniform lithium on the tips of the Cu‐wires. The LP current and time were set at 8 µA and 1 h, respectively. The potentials of the WE and CE to RE during plating lithium are shown in **Figure** **11**a,b. The incremental capacity (IC) and EIS curves of self‐made three‐electrode batteries coincide with the original batteries (Figure 11c,d) for NCM532 cells. The detailed comparison results of IC and EIS for NCM111 three‐electrode and full cells can be found in the work of Ruan et al.^[^ ^44^ ^]^ These identical results mean the successful installation of the reference electrode.

### Electrochemical Tests

All the electrochemical tests were conducted using EIS tests on the fabricated three‐electrode batteries at various SOCs and temperatures. The battery underwent a full charge process, beginning with a constant current of 0.5 C until reaching 4.2 V, followed by a constant voltage of 4.2 V until 0.05 C. Subsequently, EIS tests were performed and allowed to stabilize for 40 min before a potentiostatic EIS test at 100% SOC. Following this, the battery was discharged at 0.1 C for NCM111 cells and 0.2 C for NCM532 cells until reaching the target SOC, 10% SOC for NCM111 cells and 5% SOC for NCM532 cells, and PEIS tests after 1 h rest were conducted in cycles until 0% SOC. The test conditions included temperatures of 0, 10, and 25 °C for NCM111 cells, and temperatures of ‐10, 0, 10, 25, and 40 °C for NCM532 cells. The AC amplitude in the PEIS test was set at 5 mV over the frequency range from 10 kHz to 10 mHz, with 12 points per decade.

## Conflict of Interest

The authors declare no conflict of interest.

## Supporting information



Supporting Information

## Data Availability

The data that support the findings of this study are available from the corresponding author upon reasonable request.
