# Supplementary material for: Cross‐Scale Decoupling Kinetic Processes in Lithium‐Ion Batteries Using the Multi‐Dimensional Distribution of Relaxation Time
Source: Adv Sci (Weinh). 2024 Oct 8;11(44):2406934. doi: 10.1002/advs.202406934 (PMC11600237; doi:10.1002/advs.202406934)
Supplement: Supplementary file 1 — Supporting Information [file ADVS-11-2406934-s001.pdf]

ADVANCED SCIENCE
Open Access

Supporting Information

for *Adv. Sci.*, DOI 10.1002/advs.202406934

Cross-Scale Decoupling Kinetic Processes in Lithium-Ion Batteries Using the Multi-Dimensional Distribution of Relaxation Time

Xue Cai, Caiping Zhang, Haijun Ruan, Zeping Chen, Linjing Zhang, Dirk Uwe Sauer and Weihan Li**

Supporting Information

Cross-scale decoupling kinetic processes in lithium-ion batteries using multi-dimensional distribution of relaxation time

Xue Cai, Caiping Zhang, Haijun Ruan, Zeping Chen, Linjing Zhang, Dirk Uwe Sauer, Weihan Li***

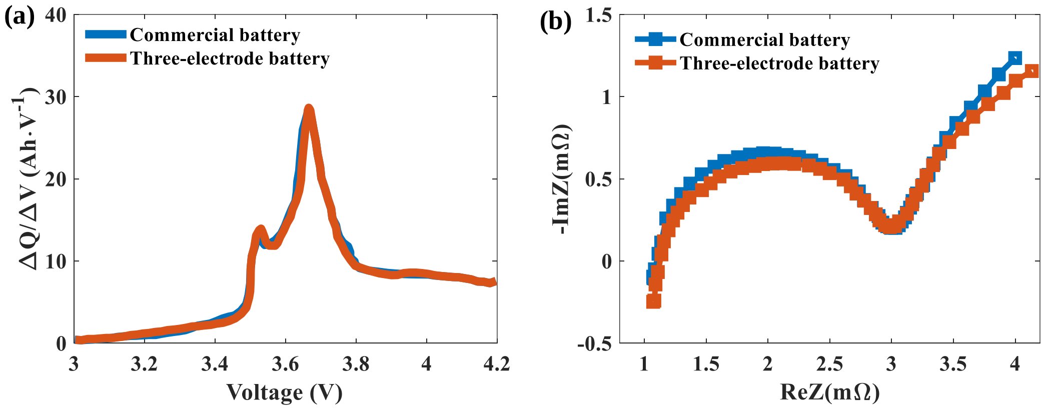

Figure S1. (a) IC curves (b) EIS at 50% SOC of the commercial and three-electrode battery.

Table. S1 The fitting results of IM at different temperatures and SOCs

25 °C RMSE (mΩ)				10 °C RMSE (mΩ)				0 °C RMSE (mΩ)			
SOC	PE	NE	FC	SOC	PE	NE	FC	SOC	PE	NE	FC
100.	0.08	0.08	0.10	100	0.08	0.13	0.13	—	—	—	—
90.4	0.06	0.10	0.12	92.8	0.10	0.12	0.13	86.9	0.08	0.30	0.28
80.8	0.07	0.09	0.10	82.7	0.08	0.14	0.13	76.0	0.09	0.32	0.26
71.2	0.07	0.09	0.09	71.6	0.08	0.13	0.14	65.3	0.09	0.31	0.27
61.5	0.07	0.08	0.10	62.4	0.09	0.15	0.15	54.0	0.10	0.31	0.31
51.9	0.08	0.07	0.09	52.1	0.09	0.15	0.14	35.4	0.08	0.31	0.27
42.3	0.07	0.10	0.10	42.0	0.07	0.12	0.15	23.5	0.10	0.32	0.31
32.7	0.07	0.11	0.11	29.5	0.08	0.12	0.16	18.3	0.11	0.30	0.30
23.1	0.07	0.12	0.14	21.3	0.10	0.12	0.16	11.2	0.11	0.31	0.30
13.5	0.07	0.11	0.16	4.5	0.24	0.22	0.22	6.2	0.13	0.29	0.33
4.8	0.11	0.16	0.25								

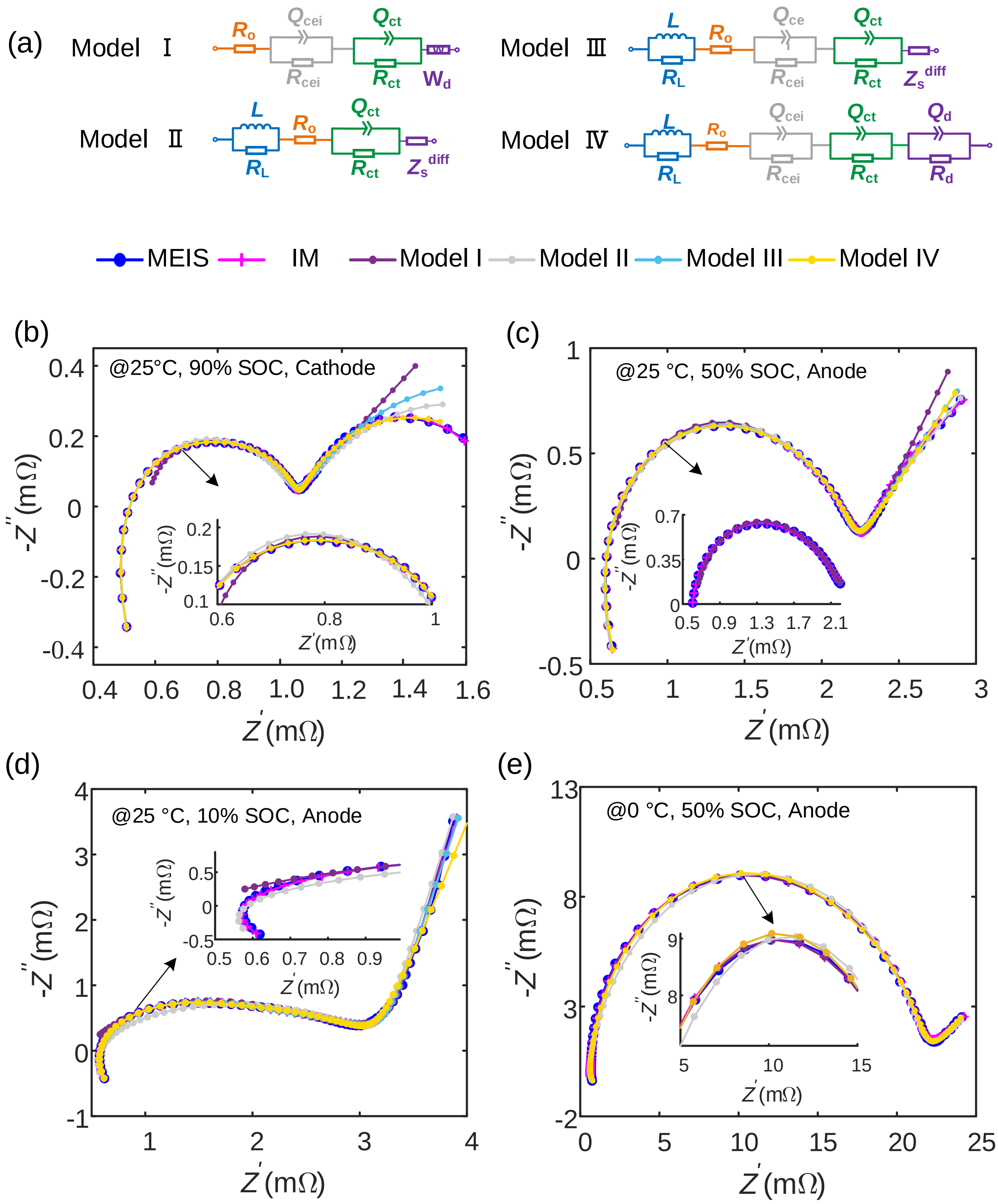

Figure S2. The fitted IM model for three-electrode batteries. (a) Equivalent circuit models. (b) to (c) Comparison of fitted results based on different models. (b) @ 25 ℃, 90% SOC, Cathode. (c) @ 25 ℃, 90% SOC, Anode. (d) @ 25 ℃, 10% SOC, Anode. (c) @ 0 ℃, 50% SOC, Anode.

To simplify the dynamic response of the liquid phase diffusion concentration (LPD) in each region in general characteristics, the average volume of the porous structure and the dimensionless normalization technique are introduced, and a general new frequency-domain concentration response equation for LPD in each region was proposed without the need of

solving the complex coefficient matrix of the partial differential equation. A simplified LPD frequency-domain expression based on three assumptions was presented:

- Fixed electrochemical parameters and collectivization in each region;
- The exchange current density is uniformly distributed in each domain and space;
- The increment of the concentration integral in the negative region equals the increment in the positive region.

For simplicity, dimensionless lengths are introduced in each reaction region:

$$\begin{cases} x_n = \dfrac{x}{L_n} (0 \le x \le L_n) \\ x_{sep} = \dfrac{x - L_n}{L_{sep}} (L_n \le x \le L_{sep}) \\ x_{p=} = \dfrac{L - x}{L_p} (L_n + L_{sep} \le x \le L) \end{cases} \tag{S1}$$

where L is thickness. The subscripts n, p, and sep represent the anode, cathode, and separator.

The liquid-phase diffusive concentration distribution in each region of the P2D model is differentiated as follows.

$$\begin{cases} \varepsilon_{e,n} \dfrac{\partial}{\partial t} c_{e,n}(x_n, t) = \dfrac{D_{e,n}^{eff}}{L_n^2} \dfrac{\partial^2 c_{e,n}(x_n, t)}{\partial x_n{}^2} + (1 - t_+) \dfrac{j_f(x_n, t)}{F} \\ \varepsilon_{e,sep} \dfrac{\partial}{\partial t} c_{e,sep}(x_{sep}, t) = \dfrac{D_{e,sep}^{eff}}{L_{sep}^2} \dfrac{\partial^2 c_{e,sep}(x_{sep}, t)}{\partial x_{sep}{}^2} \\ \varepsilon_{e,p} \dfrac{\partial}{\partial t} c_{e,p}(x_p, t) = \dfrac{D_{e,p}^{eff}}{L_p^2} \dfrac{\partial^2 c_{e,p}(x_p, t)}{\partial x_p{}^2} + (1 - t_+) \dfrac{j_f(x_p, t)}{F} \end{cases} \tag{S2}$$

where ε_e is the porosity, D_e is the liquid-phase diffusion coefficient. The superscript *eff* represents effective value. c_e is liquid-phase diffusion concentration. t_+ is the transference number of lithium cations.

The electrolyte concentration distribution at the positive and negative collector exhibits no concentration gradient along the thickness direction, and the equation can be obtained:

$$\frac{1}{L_n} \frac{\partial c_{e,n}(x_n, s)}{\partial x_n} \bigg|_{x_n = 0} = 0, \frac{1}{L_p} \frac{\partial c_{e,p}(x, s)}{\partial x_p} \bigg|_{x_p = 0} = 0 \tag{S3}$$

The electrolyte concentration distribution at the junction of the negative/positive electrode and separator is continuous and written as follows:

$$c_{e,n}(x_n, s)\big|_{x_n = 1} = c_{e,sep}(x_{sep}, s)\big|_{x_{sep} = 0} \tag{S4}$$

$$c_{e,p}(x_n, s)\big|_{x_p = 1} = c_{e,sep}(x_{sep}, s)\big|_{x_{sep} = 1} \tag{S5}$$

The concentration gradient of electrolyte concentration distribution at the junction of the negative/positive electrode and separator is continuous along the thickness direction is continuous and given as:

$$-\frac{D_{e,n}^{eff}}{L_n}\frac{\partial c_{e,n}(x,s)}{\partial x_n}\bigg|_{x_n=1} = -\frac{D_{e,sep}^{eff}}{L_{sep}}\frac{\partial c_{e,s}(x,s)}{\partial x_{sep}}\bigg|_{x_{sep}=0} \tag{S6}$$

$$-\frac{D_{e,sep}^{eff}}{L_{sep}}\frac{\partial c_{e,sep}(x,s)}{\partial x_{sep}}\bigg|_{x_{sep}=1} = -\frac{D_{e,p}^{eff}}{L_p}\frac{\partial c_{e,p}(x,s)}{\partial x_p}\bigg|_{x_p=1} \tag{S7}$$

Assuming an initial concentration of t=0, the partial differential of the liquid phase diffusion concentration distribution is transformed into an ordinary differential equation by the Rasch transform in the s-domain:

$$sc_{e,n}(x_n,s)-c_{e,n}(x_n,0)=\frac{D_{e,n}^{eff}}{\varepsilon_{e,n}L_n^2}\frac{d^2c_{e,n}(x_n,s)}{dx_n{}^2}+(1-t_0^+)\frac{I(s)}{AL_nF\varepsilon_{e,n}} \tag{S8}$$

$$sc_{e,sep}(x,s)-c_{e,sep}(x,0)=\frac{D_{e,sep}^{eff}}{\varepsilon_{e,sep}L_{sep}^2}\frac{d^2c_{e,sep}(x_{sep},t)}{dx_{sep}{}^2} \tag{S9}$$

$$sc_{e,p}(x_p,s)-c_{e,p}(x_p,0)=\frac{D_{e,p}^{eff}}{\varepsilon_{e,p}L_p^2}\frac{d^2c_{e,p}(x_p,s)}{dx_p{}^2}+(1-t_0^+)\frac{I(s)}{AL_pF\varepsilon_{e,p}} \tag{S10}$$

After introducing the dimensionless length, it is first assumed that the distribution of electrolyte ions in the positive and negative electrode and diaphragm regions conforms to the following equation:

$$\begin{cases} c_{e,n}(x_n,s)=a_n(s)x_n^2+c_n(s),(0\le x_n\le 1) \\ c_{e,sep}(x_{sep},s)=b_{sep}(s)x_{sep}+c_{sep}(s),(0\le x_{sep}\le 1) \\ c_{e,p}(x_p,s)=a_p(s)x_p^2+c_p(s),(0\le x_p\le 1) \end{cases} \tag{S11}$$

According to the liquid-phase ion distribution in each region (Equation S11), the relationship between the liquid-phase lithium-ion concentration and the polynomial coefficients for the positive-negative and positive-reference electrodes and the negative-reference electrodes can be deduced as follows:

$$\begin{cases} c_{e,n}^{diff}(s)=c_{e,n}(x_n,s)\big|_{x_n=1}-c_{e,n}(x_n,s)\big|_{x_n=0}=a_n(s) \\ c_{e,p}^{diff}(s)=c_{e,p}(x_p,s)\big|_{x_p=0}-c_{e,p}(x_p,s)\big|_{x_p=1}=-a_p(s) \\ c_{e,n-sep}^{diff}(s)=c_{e,n}(x_n,s)\big|_{x_n=1}-c_{e,sep}(x_{sep},s)\big|_{x_{sep}=0.5}=\frac{1}{2}b_{sep}(s)+a_n(s) \\ c_{e,p-sep}^{diff}(s)=c_{e,p}(x_p,s)\big|_{x_p=1}-c_{e,sep}(x_{sep},s)\big|_{x_{sep}=0.5}=\frac{1}{2}b_{sep}(s)-a_p(s) \end{cases} \tag{S12}$$

The multinomial expression in Equation S11 for each region is obtained by substituting it into the ordinary differential equation expression (4):

$$a_n(s) = \frac{D_{e,sep}^{eff} L_n}{2D_{e,n}^{eff} L_{sep}} b_{sep}(s) \tag{S13}$$

$$a_p(s) = -\frac{D_{e,sep}^{eff} L_p}{2D_{e,p}^{eff} L_{sep}} b_{sep}(s) \tag{S14}$$

$$a_n(s) + c_n(s) = c_{sep}(s) \tag{S15}$$

$$a_p(s) + c_p(s) = b_{sep}(s) + c_{sep}(s) \tag{S16}$$

The multinomial expression in Equation S5 for each region is obtained by substituting it into the ordinary differential equation expression (4):

$$sc_{e,n}(x_n, s) - c_{e,n}(x_n, 0) = \frac{2D_{e,n}^{eff}}{\varepsilon_{e,n} L_n^2} a_n(s) + (1 - t_0^+) \frac{I(s)}{AL_n F \varepsilon_{e,n}} \tag{S17}$$

$$sc_{e,sep}(x, s) - c_{e,sep}(x, 0) = 0 \tag{S18}$$

$$sc_{e,p}(x_p, s) - c_{e,p}(x_p, 0) = \frac{2D_{e,p}^{eff}}{\varepsilon_{e,p} L_p^2} a_p(s) + (1 - t_0^+) \frac{I(s)}{AL_p F \varepsilon_{e,p}} \tag{S19}$$

Subtracting Equation S15 and S16 and combining the results of subtracting Equation S13 and S14, gives:

$$c_p(s) - c_n(s) = \left(1 + \frac{D_{e,sep}^{eff} L_p}{2D_{e,p}^{eff} L_{sep}} + \frac{D_{e,sep}^{eff} L_n}{2D_{e,n}^{eff} L_{sep}} \right) b_{sep}(s) \tag{S20}$$

The liquid phase concentration difference at the positive and negative collector can be obtained from the integral expression of the liquid phase potential distribution:

$$c_{e,con}^{diff}(s)s = c_{e,p}(x_p, s)\Big|_{x_p=0} - c_{e,n}(x_n, s)\Big|_{x_n=0} = c_p(s) - c_n(s) \tag{S21}$$

Subtracting Equation S8 and S10 from the initial concentration at steady state and combining Equation S13 and S14, we obtain the expressions for the frequency-domain response of liquid-phase concentration diffusion in the positive and negative regions:

$$\begin{cases} \dfrac{c_p^{diff}(s)}{I(s)} = \dfrac{1 - t_0^+}{AL_p F \varepsilon_{e,p}} \dfrac{1}{s + \dfrac{2D_{e,p}^{eff}}{\varepsilon_{e,p} L_p^2}} \\ \dfrac{c_n^{diff}(s)}{I(s)} = \dfrac{1 - t_0^+}{AL_n F \varepsilon_{e,n}} \dfrac{1}{s + \dfrac{2D_{e,n}^{eff}}{\varepsilon_{e,n} L_n^2}} \end{cases} \tag{S22}$$

To verify the validity of the proposed liquid-phase concentration-diffusion expression, Equation S22 is compared with the analytical expression derived in the literature [1,2], and it is found that the proposed simplified equation for the liquid-phase concentration-diffusion and the Nyquist and Bode plots of the analytical expression coincide almost precisely from the frequency interval of 10^4 Hz ~ 10^{-4} Hz, and the maximum phase angle error occurs at 10^1 Hz ~ 10^0 Hz, which indicates that the proposed simplified method can express the frequency domain

characteristics of liquid-phase concentration-diffusion without loss of accuracy, and is simple and effective. From Equation S22, it is easy to see that the frequency-domain characteristics of the liquid-phase concentration-diffusion in the electrode region can be approximated as a first-order inertial link, as shown in the simplified and analytical expressions of the liquid-phase concentration-diffusion in the negative area of the Nyquist (Figure S3a), both of which are presented in the shape of a semi-circular arc. When the CPE element is of fractional order α, its amplitude-frequency characteristic has a slope of -20 α dB/decade. Taking the eigenfrequency as the turning point, the amplitude-frequency characteristic is transformed from -20dB/decade to 0 dB/decade, and its phase characteristic is transformed from -90 ℃ to 0 ℃, as shown in Figure S3b, which is the amplitude-phase characteristic of the typical first-order inertial link. Equation S22 is derived from the concentration partial differential equation for liquid-phase diffusion and its boundary conditions. It determines the simplified form of the liquid-phase diffusion frequency domain and the exact expression that can reflect the characteristic time of liquid-phase diffusion. The assumption that the electrolyte is usually neglected in most previous studies is not always correct, and the influence of the liquid-phase diffusion impedance arises from the dynamic electrochemical polarization due to the concentration difference and the ohmic polarization due to the potential difference. The characteristic time of the diffusion process is usually described as [3], where L_d and D_d are the diffusion length and diffusion coefficient, respectively. The actual liquid phase diffusion characteristic time is, where ε_i is the liquid phase volume fraction of electrode i. From the characteristic time expression, it can be seen that the liquid phase diffusion characteristic time in the electrode region is proportional to the liquid phase diffusion length and liquid phase volume fraction and inversely proportional to the liquid phase diffusion coefficient, with the proportionality coefficient of 1/2.

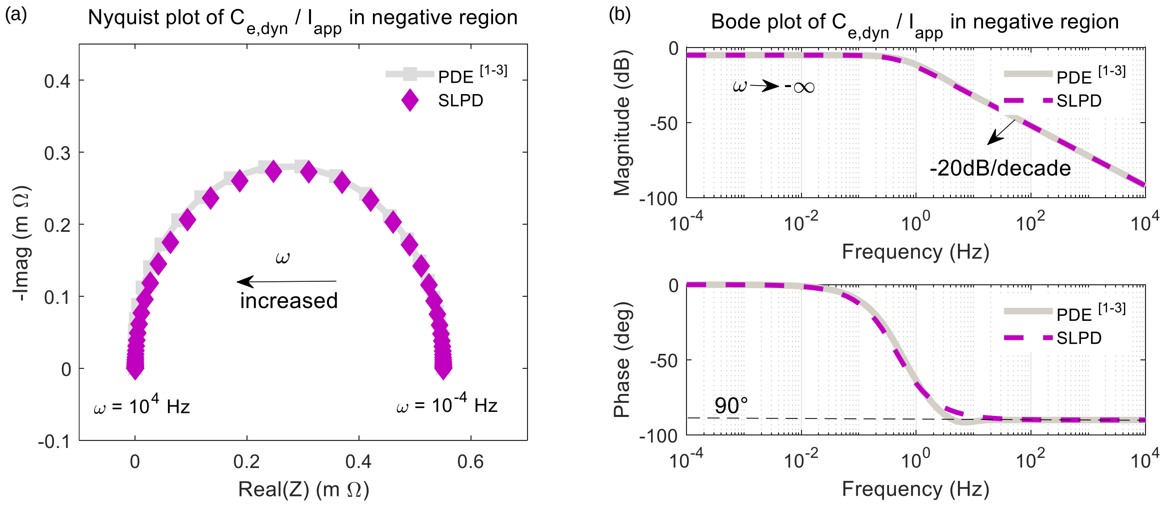

Figure S3. Simplified and analytical expressions for liquid-phase concentration diffusion in the negative region. (a) Nyquist and (b) Bode plot.

To achieve accurate modeling of the low-frequency diffusion impedance of the three-electrode cell, we introduced the concentration of the reference electrode to derive the correct WE-RE-CE LPD concentration between the two electrodes to ensure that the simplified model can characterize the low-frequency diffusion impedance without loss of accuracy. We subtract Equation S17 and S18 in the above equation and combine them with Equation S13 to obtain the concentration diffusion impedance of the working electrode WE to the reference electrode RE:

$$\frac{c_{p-sep}^{diff}(s)}{I(s)}=\frac{1-t_0^+}{A_pL_pF\varepsilon_{e,p}}\frac{1}{s+\frac{2D_{e,p}^{eff}}{\varepsilon_{e,p}L_p}(\frac{\varepsilon_{e,p}^{brugg}}{\varepsilon_{e,sep}^{brugg}}L_{sep}+L_p)} \quad \text{(S23)}$$

Similarly, we subtract Equation S19 from S18 and combine Equation S18 to obtain the concentration diffusion impedance of the counter electrode CE to the reference electrode RE:

$$\frac{c_{n-sep}^{diff}(s)}{I(s)}=\frac{1-t_0^+}{A_nL_nF\varepsilon_{e,n}}\frac{1}{s+\frac{2D_{e,n}^{eff}}{\varepsilon_{e,n}L_n}(\frac{\varepsilon_{e,n}^{brugg}}{\varepsilon_{e,sep}^{brugg}}L_{sep}+L_n)} \quad \text{(S24)}$$

Likewise, we subtract Equation S19 with Equation S17 and substitute Equation S17, S18, and S20 to obtain the concentration diffusion impedance for the working electrode WE against the reference electrode CE:

$$\frac{c_{n-p}^{diff}(s)}{I(s)}=\frac{\frac{1-t_0^+}{A_pL_pF\varepsilon_{e,p}}+\frac{1-t_0^+}{A_nL_nF\varepsilon_{e,n}}}{s+\frac{D_{e,sep}^{eff}}{L_{sep}}\frac{\frac{1}{\varepsilon_{e,p}L_p}+\frac{1}{\varepsilon_{e,n}L_n}}{1+\frac{\varepsilon_{e,sep}^{brugg}L_p}{2\varepsilon_{e,p}^{brugg}L_{sep}}+\frac{\varepsilon_{e,sep}^{brugg}L_n}{2\varepsilon_{e,n}^{brugg}L_{sep}}}} \quad \text{(S25)}$$

The effect of the electrolyte Li-ion concentration gradient on the cell terminal voltage is most directly reflected in the liquid-phase potential difference. Due to the input of a small AC signal perturbation within EIS, the starting cross-section concentration is minimal compared to the steady-state concentration, and the relationship between the two is satisfied by ζ, where ζ is a constant close to 1. A first-order Taylor equation integrates the liquid-phase potential distribution equation at any location x along the thickness direction. After integrating the equation for the distribution of the liquid-phase potential at any location x along the thickness direction, the impedance response of the liquid-phase concentration overpotential due to the gradient of electrolyte concentration between any cross-section of interest and within a three-electrode cell is derived by linearizing the equation with a first-order Taylor expansion:

$$\eta_e(s) = \xi \cdot \kappa_D^{eff} \ln\left(\frac{c_e(x_{end}, s)}{c_e(x_{start}, s)}\right) \approx \xi \cdot \kappa_D^{eff} \frac{c_{e,x}^{diff}}{c_{e,0}} \tag{S26}$$

where $\xi = (1 - t_0^+)(1-\beta)\frac{2RT}{F}$. $c_{e,0}$ is the initial electrolyte concentration and κ_D^{eff} is effective ionic conductivity. Therefore, the liquid-phase concentration difference over the potential difference caused by the liquid-phase concentration difference behaves as a first-order inertial link in the frequency domain.

References

[1] Xu J, Wang T, Pei L, et al. Parameter identification of electrolyte decomposition state in lithium-ion batteries based on a reduced pseudo two-dimensional model with Padé approximation[J]. Journal of Power Sources, 2020, 460: 228093.

[2] Yuan S, Jiang L, Yin C, et al. A transfer function type of simplified electrochemical model with modified boundary conditions and Padé approximation for Li-ion battery: Part 1. lithium concentration estimation[J]. Journal of Power Sources, 2017, 352: 245-257.

[3] Huang J, Li Z, Zhang J, et al. An analytical three-scale impedance model for porous electrode with agglomerates in lithium-ion batteries[J]. Journal of The Electrochemical Society, 2015, 162(4): A585.
